# An updated checklist of aquatic plants of Myanmar and Thailand

**DOI:** 10.3897/BDJ.2.e1019

**Published:** 2014-01-06

**Authors:** Yu Ito, Anders S. Barfod

**Affiliations:** † University of Canterbury, Christchurch, New Zealand; ‡ Aarhus University, Aarhus, Denmark

**Keywords:** Aquatic plants, flora, Myanmar, Thailand

## Abstract

The flora of Tropical Asia is among the richest in the world, yet the actual diversity is estimated to be much higher than previously reported. Myanmar and Thailand are adjacent countries that together occupy more than the half the area of continental Tropical Asia. This geographic area is diverse ecologically, ranging from cool-temperate to tropical climates, and includes from coast, rainforests and high mountain elevations. An updated checklist of aquatic plants, which includes 78 species in 44 genera from 24 families, are presented based on floristic works. This number includes seven species, that have never been listed in the previous floras and checklists. The species (excluding non-indigenous taxa) were categorized by five geographic groups with the exception of to reflect the rich diversity of the countries' floras.

## Introduction

Myanmar and Thailand, adjacent countries, comprise more than half of the tropical region for continental Asia. Situated within the Indo-Myanmar (Burma) hot-spot, this area is among the richest in biodiversity in the World (Van Dijk et al. 2004), with the estimated biodiversity being much higher than has been previously reported. The area is geographically diverse, going from cool-temperate to tropical and from the coast to rainforests to high mountains (Kress et al. 2003).

The aquatic floras of Myanmar and Thailand have been separately investigated. The Flora of Thailand project was launched in the 70’s, led by European and Thai herbaria and institutions. The aquatic species were treated in separated volumes (Table 1). The aquatic flora of Myanmar has not been published yet, although many of the species investigated are listed in the aquatic flora of India (Cook 1996).

## Materials and methods

**Figure 1.**
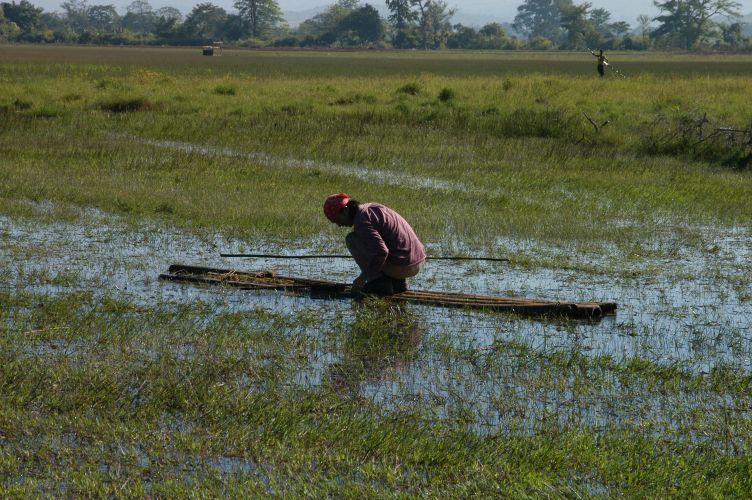
Collecting aquatic plants in a lake (Myanmar).

The aquatic plant specimens treated in this study come from field works carried out in 2006 (Myanmar), 2008 (Myanmar), and 2012 (Thailand). Plants were hand-collected from aquatic environments e.g. ponds and lakes. Occasionally collections were made by boat (Fig. 1). In addition to field collections, exhaustive herbarium surveys were conducted in major herbaria, including AAU (Denmark), BFK (Thailand), GH (USA), MBK (Japan), TI (Japan), and TNS (Japan). The herbarium specimens were morphologically identified by using Cook (1996) or the Flora of Thailand series (Table 1). For Potamogetonaceae, in addition to the above mentioned literature, Flora of China treatment was also referred (Guo et al. 2010); for Menyanthaceae, for which no volumes for the family has been published in the Flora of Thailand, Cook (1996), Ho and Ornduff (1995), and Tippery et al. (2009) were used for identification. The identified species were classified and ordered following Haston et al. (2009). Some specimens of species cited in previous floras were examined. Species distribution was obtained from regional floras. Where distribution data were absent or where the floras have not yet been published, question marks have been inserted, e.g., ?Myanmar.

## Checklists

### A checklist of aquatic plants of Myanmar and Thailand (and adjoining area)

Classification: Angiospermae

#### Nymphaeales

#### Cabombaceae

#### Cabomba

Aubl., 1775

#### Cabomba caroliniana

A. Gray., 1837

##### Distribution

Native to the Americas.

#### Nymphaeaceae

#### Barclaya

Wall., 1827

#### Barclaya longifolia

Wall., 1827

##### Materials

- **Type status:** Other material. **Occurrence:** recordedBy: Y. Ito; **Location:** country: Thailand; locality: Khao San Yot Natl Park.; verbatimLatitude: 12° 9' 15" N; verbatimLongitude: 99° 58' 44" E; **Event:** eventDate: Nov. 13, 2012; **Record Level:** collectionID: Y. Ito 1706; institutionCode: BKF
- **Type status:** Other material. **Occurrence:** recordedBy: Y. Ito; **Location:** country: Thailand; locality: Phang Nga Province; Kuraburi District, Bangwan stream; verbatimLatitude: 9° 13' 34"N; verbatimLongitude: 98° 26' 22"E; **Event:** eventDate: Oct. 25, 2006; **Record Level:** collectionID: T. Muadsud 137; institutionCode: BKF
- **Type status:** Other material. **Occurrence:** recordedBy: Y. Ito; **Location:** country: Thailand; locality: Nong Khai Province; Bungkhla District, Phu Wua Wilflife Sanctuary; verbatimLatitude: 18° 14' 41" N; verbatimLongitude: 103° 57' 58" E; **Event:** eventDate: Aug. 27, 2001; **Record Level:** collectionID: R. Pooma et al. 2781; institutionCode: BKF
- **Type status:** Other material. **Occurrence:** recordedBy: Y. Ito; **Location:** country: Thailand; locality: Kanchanaburi Province; Sangkla Buri, Nong Lu, Tham kaeo Sawan Bandan Temple; verbatimLatitude: 15° 16' 20" N; verbatimLongitude: 98° 28' 3" E; **Event:** eventDate: Aug. 25, 2010; **Record Level:** collectionID: V. Chamchumroon et al. 4807; institutionCode: BKF
- **Type status:** Other material. **Occurrence:** recordedBy: Y. Ito; **Location:** country: Thailand; locality: Nong khai Province; Bungklaa, Phutoknoi; verbatimLatitude: 17° 56' 32" N; verbatimLongitude: 102° 44' 57" E; **Event:** eventDate: Jun. 21, 1997; **Record Level:** collectionID: C. Niyomdham 5090; institutionCode: AAU
- **Type status:** Other material. **Occurrence:** recordedBy: Y. Ito; **Location:** country: Thailand; locality: Ubon Ratchathani Province; Soi Sawan waterfall; verbatimLatitude: 15° 27' 35" N; verbatimLongitude: 105° 34' 44" E; **Event:** eventDate: Nov. 15, 2003; **Record Level:** collectionID: W. La-ongsri 24269; institutionCode: BKF
- **Type status:** Other material. **Occurrence:** recordedBy: Y. Ito; **Location:** country: Thailand; locality: Ubon Ratchatani Province; Khong Chian District, Udob ratehatani Gene conservation station; verbatimLatitude: 15° 27' 1" N; verbatimLongitude: 105° 29' 2" E; **Event:** eventDate: Sep. 15, 2001; **Record Level:** collectionID: J.F. Maxwell 01-436; institutionCode: GH
- **Type status:** Other material. **Occurrence:** recordedBy: Y. Ito; **Location:** country: Thailand; locality: Nong Khai Province; Phn Wun Wildlife Sanctuary, Thamtonn fall; verbatimLatitude: 17° 53' 7" N; verbatimLongitude: 102° 45' 2" E; **Event:** eventDate: Jun. 14, 2004; **Record Level:** collectionID: Th. Wongprasert et al. 046-85; institutionCode: BKF
- **Type status:** Other material. **Occurrence:** recordedBy: Y. Ito; **Location:** country: Thailand; locality: Saraburi Province; Salum Lake; verbatimLatitude: 14° 32' N; verbatimLongitude: 101° 2' E; **Event:** eventDate: Oct. 14, 1973; **Record Level:** collectionID: J.F. Maxwell 73-503; institutionCode: AAU
- **Type status:** Other material. **Occurrence:** recordedBy: Y. Ito; **Location:** country: Thailand; locality: Chantaburi Province; S of Mekam rubber plantation; verbatimLatitude: 12° 35' N; verbatimLongitude: 102° 20' E; **Event:** eventDate: Aug. 22, 1966; **Record Level:** collectionID: K. Larsen et al. 1746; institutionCode: AAU
- **Type status:** Other material. **Occurrence:** recordedBy: Y. Ito; **Location:** country: Thailand; locality: Phangnga Province; Takuapah District, 15 km N of Takuapah; verbatimLatitude: 8° 53' N; verbatimLongitude: 98° 21' E; **Event:** eventDate: Jul. 13, 1972; **Record Level:** collectionID: K. Larsen et al. 30887; institutionCode: AAU

##### Distribution

India (Southern [Andaman Isl.]), ?Myanmar, Thailand.

##### Notes

Fig. 2.

#### Nymphaea

L., 1753

#### Nymphaea nouchali

Burm. f., 1768

##### Materials

- **Type status:** Other material. **Occurrence:** recordedBy: Y. Ito; **Location:** country: Myanmar; locality: Bago Division; border to Rakhain State; verbatimLatitude: 18° 40' 27'' N; verbatimLongitude: 94° 53' 41'' E; **Event:** eventDate: Dec. 11, 2006; **Record Level:** collectionID: Sugawara et al. 036514; institutionCode: TI
- **Type status:** Other material. **Occurrence:** recordedBy: Y. Ito; **Location:** country: Myanmar; locality: Rakhain State; between God village and mangrove forest; verbatimLatitude: 18° 29' 30" N; verbatimLongitude: 94° 16' 13" E; **Event:** eventDate: Dec. 12, 2006; **Record Level:** collectionID: Sugawara et al. 036535; institutionCode: TI
- **Type status:** Other material. **Occurrence:** recordedBy: Y. Ito; **Location:** country: Thailand; locality: Sattalip, Taong Breng; verbatimLatitude: 12° 43' N; verbatimLongitude: 100° 56' E; **Event:** eventDate: Oct. 1, 1969; **Record Level:** collectionID: J.F. Maxwell s.n.; institutionCode: AAU
- **Type status:** Other material. **Occurrence:** recordedBy: Y. Ito; **Location:** country: Thailand; locality: Narathiwat Province; S of Naratiwat; verbatimLatitude: 6° 30' N; verbatimLongitude: 101° 45' E; **Event:** eventDate: Mar. 8, 1974; **Record Level:** collectionID: K. Larsen & S.S. Larsen 33086; institutionCode: AAU
- **Type status:** Other material. **Occurrence:** recordedBy: Y. Ito; **Location:** country: Thailand; locality: Ayuthaya Province; Ayuthaya District; verbatimLatitude: 14° 22' N; verbatimLongitude: 100° 35' E; **Event:** eventDate: Dec. 3, 1979; **Record Level:** collectionID: T. Shimizu et al. T-26093; institutionCode: AAU
- **Type status:** Other material. **Occurrence:** recordedBy: Y. Ito; **Location:** country: Thailand; locality: Phetchabury; verbatimLatitude: 13° 30' 06" N; verbatimLongitude: 99° 47' 37" E; **Event:** eventDate: Nov. 14, 2012; **Record Level:** collectionID: Y. Ito 1711; institutionCode: BKF

##### Distribution

Bangladesh, China (Southern), India (nationwide), ?Indonesia, Myanmar, Pakistan, Papua New guinea, ?Philippines, Thailand, Sri Lanka, ?Vietnam.

#### Nymphaea pubescens

Willd., 1799

##### Materials

- **Type status:** Other material. **Occurrence:** recordedBy: Y. Ito; **Location:** country: Myanmar; locality: Inle lake; verbatimLatitude: 20° 35' 21" N; verbatimLongitude: 96° 54' 34" E; **Event:** eventDate: May. 1, 1938; **Record Level:** collectionID: F. G. Dickason 7867; institutionCode: GH
- **Type status:** Other material. **Occurrence:** recordedBy: Y. Ito; **Location:** country: Myanmar; locality: Bago Division; W of Pyay; verbatimLatitude: 18° 42' 22" N; verbatimLongitude: 95° 5' 59" E; **Event:** eventDate: Dec. 9, 2006; **Record Level:** collectionID: Sugawara et al. 036431; institutionCode: TI
- **Type status:** Other material. **Occurrence:** recordedBy: Y. Ito; **Location:** country: Thailand; locality: Rachaburi Province; Huai Yang waterfall; verbatimLatitude: 12° 29' N; verbatimLongitude: 99° 41' E; **Event:** eventDate: Aug. 14, 1966; **Record Level:** collectionID: K. Larsen et al. 1588; institutionCode: AAU
- **Type status:** Other material. **Occurrence:** recordedBy: Y. Ito; **Location:** country: Thailand; locality: Mae Sariang Province; Ban Huai Sai 12 km A of Mas Sariang; verbatimLatitude: 18° 6' N; verbatimLongitude: 97° 55' E; **Event:** eventDate: Jul. 12, 1968; **Record Level:** collectionID: K. Larsen et al. 2370; institutionCode: AAU
- **Type status:** Other material. **Occurrence:** recordedBy: Y. Ito; **Location:** country: Thailand; locality: Phattalung Province; Khuankhanum District; Talenoi Lake, Talenoi Wildlife Sanctuary; verbatimLatitude: 7° 46' N; verbatimLongitude: 100° 7' E; **Event:** eventDate: Dec. 20, 1979; **Record Level:** collectionID: T. Shimizu et al. 27730; institutionCode: AAU
- **Type status:** Other material. **Occurrence:** recordedBy: Y. Ito; **Location:** country: Thailand; locality: Khao San Yot Natl Park.; verbatimLatitude: 12° 14' 42" N; verbatimLongitude: 99° 55' 60" E; **Event:** eventDate: Nov. 13, 2012; **Record Level:** collectionID: Y. Ito 1704; institutionCode: BKF

##### Distribution

Bangladesh, India (nationwide), Myanmar, Pakistan, Papua New Guinea, Thailand, Sri Lanka.

#### Acorales

#### Acoraceae

#### Acorus

L., 1753

#### Acorus calamus

L., 1753

##### Materials

- **Type status:** Other material. **Occurrence:** recordedBy: Y. Ito; **Location:** country: Myanmar; verbatimLatitude: 16° 53' 19" N; verbatimLongitude: 95° 52' 29" E; **Record Level:** collectionID: MBK025720; institutionCode: TI
- **Type status:** Other material. **Occurrence:** recordedBy: Y. Ito; **Location:** country: Myanmar; verbatimLatitude: 16° 53' 19.18"; verbatimLongitude: 95° 52' 28.59"; **Record Level:** collectionID: MBK032409; institutionCode: TI
- **Type status:** Other material. **Occurrence:** recordedBy: Y. Ito; **Location:** country: Myanmar; verbatimLatitude: 16° 53' 19.18"; verbatimLongitude: 95° 52' 28.59"; **Record Level:** collectionID: TI032666; institutionCode: TI
- **Type status:** Other material. **Occurrence:** recordedBy: Y. Ito; **Location:** country: Thailand; locality: Chiang Mai Province; Juuigalion - canal, S of Chiang Mai; verbatimLatitude: 20° 11' N; verbatimLongitude: 99° 46' E; **Event:** eventDate: Mar. 2, 1958; **Record Level:** collectionID: T. Sorensen et al. 1812; institutionCode: GH

##### Distribution

Worldwide.

#### Acorus gramineus

Sol. ex Aiton, 1789

##### Materials

- **Type status:** Other material. **Occurrence:** recordedBy: Y. Ito; **Location:** country: Myanmar; locality: NE Burma, Kambaiti (73 km E of Myitkyina); verbatimLatitude: 25° 24' 22" N; verbatimLongitude: 98° 8' 39" E; **Event:** eventDate: Apr. 17, 1905; **Record Level:** collectionID: R. Malaise 85; institutionCode: GH
- **Type status:** Other material. **Occurrence:** recordedBy: Y. Ito; **Location:** country: Myanmar; locality: North triangle (Hbinlum) 3000'; verbatimLatitude: 20° 21' N; verbatimLongitude: 100° 4' E; **Event:** eventDate: Apr. 30, 1953; **Record Level:** collectionID: F. Kingdon-Ward 20756; institutionCode: GH
- **Type status:** Other material. **Occurrence:** recordedBy: Y. Ito; **Location:** country: Myanmar; locality: Kachin division, Sumprabum Sub-Division, eastern approaches from Sumprabum to Kumon range.; verbatimLatitude: 26° 40' N; verbatimLongitude: 97° 20' E; **Event:** eventDate: Mar. 1, 1962; **Record Level:** collectionID: J. Keenan, U. Tun Aung, U. Tha Hla 3815; institutionCode: GH
- **Type status:** Other material. **Occurrence:** recordedBy: Y. Ito; **Location:** country: Thailand; locality: Chiang Mai Province; Muung District, Doi Sutap-Pui National Park, E side; verbatimLatitude: 18° 48' 16" N; verbatimLongitude: 98° 54' 56" E; **Event:** eventDate: Apr. 18, 1990; **Record Level:** collectionID: J.F. Maxwell 90-429; institutionCode: GH
- **Type status:** Other material. **Occurrence:** recordedBy: Y. Ito; **Location:** country: Thailand; locality: Chiang Mai Province; Doi Sutap-Pui National Park, CXU observation area; verbatimLatitude: 18° 48' 16" N; verbatimLongitude: 98° 54' 56" E; **Event:** eventDate: Jan. 7, 1993; **Record Level:** collectionID: J.F. Maxwell 93-008; institutionCode: GH
- **Type status:** Other material. **Occurrence:** recordedBy: Y. Ito; **Location:** country: Thailand; locality: Lampoon Province; Mae Tah District, Doi Kuhn Dehn National Park, stream above Pah Dtoop Fall; verbatimLatitude: 18° 30' 25" N; verbatimLongitude: 99° 16' 11" E; **Event:** eventDate: Apr. 30, 1994; **Record Level:** collectionID: J.F. Maxwell 94-547; institutionCode: GH
- **Type status:** Other material. **Occurrence:** recordedBy: Y. Ito; **Location:** country: Thailand; locality: Lampang Province; Muang Bahn District, Jae Sawn National Park, Bah Wiang village area; verbatimLatitude: 19° 9' 39" N; verbatimLongitude: 99° 23' 45" E; **Event:** eventDate: Feb. 15, 1996; **Record Level:** collectionID: J.F. Maxwell 96-227; institutionCode: GH
- **Type status:** Other material. **Occurrence:** recordedBy: Y. Ito; **Location:** country: Thailand; locality: Chiang Mai Province; Wiong Bah bao District, Kuhn Jae (Chae) national park; verbatimLatitude: 19° 10' 4" N; verbatimLongitude: 99° 23' 40" E; **Event:** eventDate: Apr. 2, 1998; **Record Level:** collectionID: J.F. Maxwell 98-362; institutionCode: GH

##### Distribution

?Cambodia, India (north), Japan, Korea, ?Laos, Myanmar, ?Philippines, Thailand, ?Vietnam.

#### Araceae

#### Aroideae

#### Cryptocoryne

Fisch. ex Wydl., 1830

#### Cryptocoryne albida

Prain, 1900

##### Materials

- **Type status:** Other material. **Occurrence:** recordedBy: Y. Ito; **Location:** country: Thailand; locality: Phang Nga province; Sri Phang Nga Natl. Park; verbatimLatitude: 8° 0' N; verbatimLongitude: 98° 28' E; **Event:** eventDate: Dec. 11, 2003; **Record Level:** collectionID: A.S. Barfod et al. 557; institutionCode: AAU
- **Type status:** Other material. **Occurrence:** recordedBy: Y. Ito; **Location:** country: Thailand; locality: Ranawng; verbatimLatitude: 10° 7' N; verbatimLongitude: 98° 52' E; **Event:** eventDate: Jan. 30, 1958; **Record Level:** collectionID: N/A 855; institutionCode: GH
- **Type status:** Other material. **Occurrence:** recordedBy: Y. Ito; **Location:** country: Thailand; locality: Phetchaburi, Amphoe kaeng Krachan, National Park.; verbatimLatitude: 12° 51' N; verbatimLongitude: 99° 18' E; **Event:** eventDate: Dec. 12, 2002; **Record Level:** collectionID: D. J. Middleton et al. 1589; institutionCode: GH
- **Type status:** Other material. **Occurrence:** recordedBy: Y. Ito; **Location:** country: Thailand; locality: Surat Thani, phanom, Khao Sok National Park.; verbatimLatitude: 8° 55' N; verbatimLongitude: 98° 31' E; **Event:** eventDate: Jan. 1, 2006; **Record Level:** collectionID: D. J. Middleton et al. 3992; institutionCode: GH

##### Distribution

?Myanmar, Thailand.

#### Cryptocoryne cordata

Griff., 1850

##### Materials

- **Type status:** Other material. **Occurrence:** recordedBy: Y. Ito; **Location:** country: Thailand; locality: Narathiwat Province; Paa Ye, Su Ngi Paadee; verbatimLatitude: 6° 8' 11" N; verbatimLongitude: 101° 54' 38" E; **Event:** eventDate: Apr. 14, 1988; **Record Level:** collectionID: C. Niyomdham & W. Ueachirakan 1820; institutionCode: AAU
- **Type status:** Other material. **Occurrence:** recordedBy: Y. Ito; **Location:** country: Thailand; locality: Narathiwat Province; Freshwater swamp-forest S of Narathiwat; verbatimLatitude: 6° 30' N; verbatimLongitude: 101° 45' E; **Event:** eventDate: Mar. 8, 1974; **Record Level:** collectionID: K. Larsen & S.S. Larsen 33077; institutionCode: AAU

##### Distribution

Malaysia (Peninsular), Thailand.

#### Cryptocoryne crispatula

Engl., 1920

##### Materials

- **Type status:** Other material. **Occurrence:** recordedBy: Y. Ito; **Location:** country: Thailand; locality: Kanchanaburi Province; Southwest District, Pompee village ner Khwae Noi river, E of Sanghkla; verbatimLatitude: 14° 1' 14" N; verbatimLongitude: 99° 19' 55" E; **Event:** eventDate: Mar. 25, 1968; **Record Level:** collectionID: C.F. van Beusekom, C. Phengkhlai 89; institutionCode: AAU
- **Type status:** Other material. **Occurrence:** recordedBy: Y. Ito; **Location:** country: Thailand; locality: Mae Hong Song, Muang District, Zuza Waterfalls, Lum Nam Pai Wildlife Sanctuary; verbatimLatitude: 19° 28' 29" N; verbatimLongitude: 98° 7' 36" E; **Event:** eventDate: Dec. 15, 2007; **Record Level:** collectionID: HN8405; institutionCode: TI
- **Type status:** Other material. **Occurrence:** recordedBy: Y. Ito; **Location:** country: Thailand; locality: Mae Hong Song, Muang District, Pha Waterfalls, Tham Pla-Namtok Pha Sua Natl Park.; verbatimLatitude: 19° 28' N; verbatimLongitude: 98° 7' E; **Event:** eventDate: Dec. 15, 2007; **Record Level:** collectionID: HN8436; institutionCode: TI
- **Type status:** Other material. **Occurrence:** recordedBy: Y. Ito; **Location:** country: Myanmar; locality: Kachin State; between Khalone Village and Shinbwiyang; verbatimLatitude: 26° 56' N; verbatimLongitude: 96° 52' E; **Event:** eventDate: Dec. 5, 2005; **Record Level:** collectionID: Murata et al. 041626; institutionCode: TI
- **Type status:** Other material. **Occurrence:** recordedBy: Y. Ito; **Location:** country: Myanmar; locality: Kachin State; between Khalone Village and Shinbwiyang; verbatimLatitude: 26° 40' 29" N; verbatimLongitude: 96° 16' 33" E; **Event:** eventDate: Dec. 5, 2005; **Record Level:** collectionID: Murata et al. 040869; institutionCode: TI

##### Distribution

Bangradesh, Cambodia, China, Laos, Myanmar, Thailand, Vietnam.

#### Cryptocoryne cruddasiana

Prain, 1900

##### Materials

- **Type status:** Other material. **Occurrence:** recordedBy: Y. Ito; **Location:** country: Myanmar; locality: S. ? G. Gumfrabum 1000' - 1500'; **Event:** eventDate: Dec. 19, 1953; **Record Level:** collectionID: F. Kingdon Ward 21712; institutionCode: GH
- **Type status:** Other material. **Occurrence:** recordedBy: Y. Ito; **Location:** country: Myanmar; locality: Kachin State; between Khalone Village and Shinbwiyang; verbatimLatitude: 26° 40' 50" N; verbatimLongitude: 96° 15' 20" E; **Event:** eventDate: Dec. 5, 2005; **Record Level:** collectionID: Murata et al. 041201; institutionCode: TI

##### Distribution

Myanmar, ?Thailand.

#### Pistia

L., 1753

#### Pistia stratiotes

L., 1753

##### Materials

- **Type status:** Other material. **Occurrence:** recordedBy: Y. Ito; **Location:** country: Thailand; verbatimLocality: Bangkok; **Event:** eventDate: Jul. 16, 1966; **Record Level:** collectionID: K. Larsen 446; institutionCode: AAU

##### Distribution

Worldwide (mainly tropics).

#### Lemnoidea

#### Landoltia

Les & D.J. Crawford, 1999

#### Landoltia punctata

(G. Mey.) Les & D.J. Crawford, 1999

##### Materials

- **Type status:** Other material. **Occurrence:** recordedBy: Y. Ito; **Location:** country: Myanmar; **Event:** eventDate: Dec. 3, 2008; **Record Level:** institutionCode: TI; collectionCode: Tanaka et al. 080648

##### Distribution

Worldwide.

#### Lemna

L., 1753

#### Lemna aequinoctialis

Welw., 1859

##### Materials

- **Type status:** Other material. **Occurrence:** recordedBy: Y. Ito; **Location:** country: Vietnam; locality: Kontum Province; Ngoc Linh mountain, Ngoc Linh village.; verbatimLatitude: 14° 20' N; verbatimLongitude: 107° 59' E; **Event:** eventDate: Apr. 4, 1995; **Record Level:** collectionID: L. Averyanov et al. VH1199; institutionCode: AAU

##### Distribution

Bangladesh, China (nationwide), India (nationwide), Japan, Myanmar, Nepal, Pakistan, Thailand; Africa; Oceania; N. America; S. America.

#### Lemna trisulca

L., 1753

##### Materials

- **Type status:** Other material. **Occurrence:** recordedBy: Y. Ito; **Location:** country: Myanmar; locality: Shan State; Inlay lake, Nyanug She Township; verbatimLatitude: 20° 32' 2" N; verbatimLongitude: 96° 53' 53" E; **Event:** eventDate: Dec. 3, 2008; **Record Level:** collectionID: Tanaka et al. 080649; institutionCode: TI

##### Distribution

Bangladesh, China (Northern, Western, Southern [Taiwan, Yunnan]), India (Eastern, Northern), Indonesia (New Guinea), Japan, Malaysia (Sumatra), Myanmar, Papua New guinea, Pakistan, Philippines; Europe; Oceania; N. America; S. America.

#### Spirodela

Schleid., 1839

#### Spirodela polyrrhiza

(L.) Schleid., 1839

##### Materials

- **Type status:** Other material. **Occurrence:** recordedBy: Y. Ito; **Location:** country: Myanmar; locality: Shan State; Inlay lake, Nyanug She Township; verbatimLatitude: 20° 32' 2" N; verbatimLongitude: 96° 53' 53" E; **Event:** eventDate: Dec. 3, 2008; **Record Level:** collectionID: Tanaka et al. 080648; institutionCode: MBK

##### Distribution

Bangladesh, China (nationwide), India (nationwide), Indonesia (Java, Sumatra, Sulawesi), Japan, Malaysia (Peninsular), Myanmar, Nepal, Pakistan, Papua New Guinea, Philippines, Thailand.

#### Wolffia

Horkel ex Schleid., 1844

#### Wolffia globosa

(Roxb.) Hartog & Plas, 1970

##### Distribution

Bangladesh, ?Cambodia, India (nationwide), ?Indonesia, Japan, ?Laos, ?Malaysia, ?Myanmar, Nepal, Pakistan, Papua New Guinea, ?Philippines, Sri Lanka, Thailand, ?Vietnam; introduced in Americas.

#### Alismatales

#### Alismataceae

#### Alisma

L., 1753

#### Alisma plantago-aquatica

L., 1753

##### Materials

- **Type status:** Other material. **Occurrence:** recordedBy: Y. Ito; **Location:** country: Myanmar; locality: Mandalay Division; Pyin U Lwin (Maymyo); **Event:** eventDate: May 1, 1932; **Record Level:** collectionID: F. G. Dickason 5798; institutionCode: GH
- **Type status:** Other material. **Occurrence:** recordedBy: Y. Ito; **Location:** country: Myanmar; locality: Shan State, Kalow; **Event:** eventDate: Nov. 26, 2008; **Record Level:** collectionID: Tanaka et al. 080047; institutionCode: TI
- **Type status:** Other material. **Occurrence:** recordedBy: Y. Ito; **Location:** country: Myanmar; locality: Shan State; Kalow; **Event:** eventDate: May 1, 1932; **Record Level:** collectionID: F. G. Dickason 5743; institutionCode: GH
- **Type status:** Other material. **Occurrence:** recordedBy: Y. Ito; **Location:** country: Myanmar; locality: Shan State; Kalow; **Event:** eventDate: May 8, 1931; **Record Level:** collectionID: F. G. Dickason; institutionCode: GH
- **Type status:** Other material. **Occurrence:** recordedBy: Y. Ito; **Location:** country: Thailand; locality: Chiang Mai; above Bong Lom Tang (Karen) village, mae Win Subdistrict; **Event:** eventDate: Mar. 21, 2003; **Record Level:** collectionID: J.F. Maxwell 03-55; institutionCode: GH
- **Type status:** Other material. **Occurrence:** recordedBy: Y. Ito; **Location:** country: Thailand; locality: Chiang Mai Province; between Ban Bo Luang and Mae Sariang; **Event:** eventDate: Jun. 13, 1968; **Record Level:** collectionID: C.F. van Beusekom, C. Phengkhlai 1200; institutionCode: AAU
- **Type status:** Other material. **Occurrence:** recordedBy: Y. Ito; **Location:** country: Thailand; locality: Chiang Mai Province; Hod District, Mai Muang Nao Arboretum, Ban Mae Sanam Mai, Baw Salee Subdistrict, 53 km Hod - Mae Sariang road (hw. 108); **Event:** eventDate: Apr. 11, 2001; **Record Level:** collectionID: W. Sankamethawee 151; institutionCode: BKF
- **Type status:** Other material. **Occurrence:** recordedBy: Y. Ito; **Location:** country: Thailand; locality: Mae Hong Son Province; Mae Sariung, Ban Papae; verbatimLatitude: 18° 54' N; verbatimLongitude: 98° 8' E; **Event:** eventDate: Feb 2, 1969; **Record Level:** collectionID: T. Leiitinand e Sa-nga 10671; institutionCode: AAU

##### Distribution

China (North-Eastern, North-Western, South [Yunnan]), Japan, Myanmar, Nepal, Thailand; Europe; Oceania; N. America.

#### Caldesia

Parl., 1860

#### Caldesia parnassifolia

(Bassi ex L.) Parl., 1858

##### Materials

- **Type status:** Other material. **Occurrence:** recordedBy: Y. Ito; **Location:** country: Laos; locality: Savannaket Province; Nakai Plateau, Theun Douan lake, near Phong Sa Vahn resettlement village.; verbatimLatitude: 16° 34' 10" N; verbatimLongitude: 104° 44' 54" E; **Event:** eventDate: May 3, 2007; **Record Level:** collectionID: J.F. Maxwell 07-305; institutionCode: GH
- **Type status:** Other material. **Occurrence:** recordedBy: Y. Ito; **Location:** country: Myanmar; locality: Shan State; Taunggyi; verbatimLatitude: 20° 47' 7" N; verbatimLongitude: 97° 2' 6" E; **Event:** eventDate: May 1, 1938; **Record Level:** collectionID: F. G. Dickason 9370; institutionCode: GH
- **Type status:** Other material. **Occurrence:** recordedBy: Y. Ito; **Location:** country: Myanmar; locality: Shan State, Pindaya; verbatimLatitude: 20° 56' 21" N; verbatimLongitude: 96° 39' 54" E; **Event:** eventDate: Dec. 1, 2008; **Record Level:** collectionID: Tanaka et al. 080624 #1; institutionCode: TI
- **Type status:** Other material. **Occurrence:** recordedBy: Y. Ito; **Location:** country: Myanmar; locality: Shan State, Pindaya; verbatimLatitude: 20° 56' 21"; verbatimLongitude: 96° 39' 54" E; **Event:** eventDate: Dec. 1, 2008; **Record Level:** collectionID: Tanaka et al. 080624 #2; institutionCode: TI

##### Distribution

Bangladesh, China (North-Eastern, South [Yunnan]), Japan, India (North, South, West), Indonesia (Sulawesi, New Guinea), Laos, Myanmar, Nepal, Papua New guinea, Thailand, ?Vietnam; Africa; Europe; Oceania.

##### Notes

Myanmar (Ito et al. 2009).

#### Limnocharis

Bonpl., 1808

#### Limnocharis flava

(L.) Buchenau, 1868

##### Materials

- **Type status:** Other material. **Occurrence:** recordedBy: Y. Ito; **Location:** country: Myanmar; locality: Rangoon.; verbatimLatitude: 16° 49' 37" N; verbatimLongitude: 96° 8' 58" E; **Event:** eventDate: Mar. 10, 1957; **Record Level:** collectionID: F. G. Dickason 6666; institutionCode: GH
- **Type status:** Other material. **Occurrence:** recordedBy: Y. Ito; **Location:** country: Myanmar; locality: Rakhine State; Bago Division; verbatimLatitude: 18° 49' 44'' N; verbatimLongitude: 95° 18' 6'' E; **Event:** eventDate: Dec. 7, 2008; **Record Level:** collectionID: Tanaka et al. 080766; institutionCode: MBK
- **Type status:** Other material. **Occurrence:** recordedBy: Y. Ito; **Location:** country: Thailand; locality: Chiang Mai; verbatimLatitude: 19° 23' N; verbatimLongitude: 99° 10' E; **Event:** eventDate: Feb. 14, 1958; **Record Level:** collectionID: Kau Cloi 731; institutionCode: AAU
- **Type status:** Other material. **Occurrence:** recordedBy: Y. Ito; **Location:** country: Thailand; locality: Sattalip, Taong Grang; verbatimLatitude: 12° 43' N; verbatimLongitude: 100° 56' E; **Event:** eventDate: Jan. 24, 1969; **Record Level:** collectionID: J.F. Maxwell; institutionCode: AAU
- **Type status:** Other material. **Occurrence:** recordedBy: Y. Ito; **Location:** country: Thailand; locality: Trang Province: Muang District; Kaochong Park; verbatimLatitude: 7° 33' 53" N; verbatimLongitude: 99° 36' 37" E; **Event:** eventDate: Feb. 3, 1985; **Record Level:** collectionID: P. Sirirugsa 990; institutionCode: AAU
- **Type status:** Other material. **Occurrence:** recordedBy: Y. Ito; **Location:** country: Thailand; locality: Trang Province; Muang District, Kao Chong Park.; verbatimLatitude: 7° 33' 53" N; verbatimLongitude: 99° 36' 37" E; **Event:** eventDate: Oct. 31, 1984; **Record Level:** collectionID: P. Sirirugsa 939; institutionCode: GH
- **Type status:** Other material. **Occurrence:** recordedBy: Y. Ito; **Location:** country: Thailand; locality: Chiang Mai Province; 30 km SW of Chiengmai.; verbatimLatitude: 19° 11' N; verbatimLongitude: 98° 4' E; **Event:** eventDate: Nov. 4, 1958; **Record Level:** collectionID: No. 6043; institutionCode: GH
- **Type status:** Other material. **Occurrence:** recordedBy: Y. Ito; **Location:** country: Thailand; locality: Petchabury; verbatimLatitude: 13° 24' 30" N; verbatimLongitude: 99° 48' 44" E; **Event:** eventDate: Nov. 14, 2012; **Record Level:** collectionID: YI1710; institutionCode: BKF

##### Distribution

Native to Americas; naturalized to tropical Asia.

#### Sagittaria

L., 1753

#### Sagittaria guayanensis

Humb., 1815

##### Materials

- **Type status:** Other material. **Occurrence:** recordedBy: Y. Ito; **Location:** country: Thailand; locality: Pathum Thani Province, Rangsit; verbatimLatitude: 13° 59' 17" N; verbatimLongitude: 100° 37' 59" E; **Event:** eventDate: Aug. 25, 1974; **Record Level:** collectionID: S. Sripen s.n.; institutionCode: BKF
- **Type status:** Other material. **Occurrence:** recordedBy: Y. Ito; **Location:** country: Thailand; locality: Mae Hong Son Province; verbatimLatitude: 19° 15' N; verbatimLongitude: 98° E; **Event:** eventDate: Sep. 9, 1974; **Record Level:** collectionID: K. Larsen & S.S. Larsen 34312; institutionCode: BKF
- **Type status:** Other material. **Occurrence:** recordedBy: Y. Ito; **Location:** country: Thailand; locality: Mae Hong Son Province; verbatimLatitude: 19° 15' N; verbatimLongitude: 98° E; **Event:** eventDate: Sep. 10, 1974; **Record Level:** collectionID: K. Larsen & S.S. Larsen 34375; institutionCode: BKF
- **Type status:** Other material. **Occurrence:** recordedBy: Y. Ito; **Location:** country: Thailand; locality: Chiang Mai; Doi Lodge; verbatimLatitude: 19° 18' N; verbatimLongitude: 99° 10' E; **Event:** eventDate: Apr. 15, 1958; **Record Level:** collectionID: No. 2742; institutionCode: GH

##### Distribution

Bangladesh, ?Cambodia, China (Central, South), India (nationwide), Indonesia (Java, Sumatra, Sulawesi), Malaysia (Peninsular), Nepal, Thailand, ?Vietnam.

#### Sagittaria trifolia

L., 1753

##### Materials

- **Type status:** Other material. **Occurrence:** recordedBy: Y. Ito; **Location:** country: Myanmar; locality: Shan State; Taungyi; verbatimLatitude: 20° 47' N; verbatimLongitude: 97° 2' E; **Event:** eventDate: May. 3, 1933; **Record Level:** collectionID: F. G. Dickason 5846; institutionCode: GH
- **Type status:** Other material. **Occurrence:** recordedBy: Y. Ito; **Location:** country: Thailand; locality: Chiang Mai; 15 km N of Chiang Mai; verbatimElevation: 18° 55' N; verbatimLatitude: 98° 57' E; **Event:** eventDate: Aug. 11, 1958; **Record Level:** collectionID: Kai Larsen 6057; institutionCode: GH
- **Type status:** Other material. **Occurrence:** recordedBy: Y. Ito; **Location:** country: Thailand; locality: Chiang Mai Province; Phek Khaeng Kai; verbatimLatitude: 18° 47' 30" N; verbatimLongitude: 98° 57' 39" E; **Event:** eventDate: Feb. 20, 1991; **Record Level:** collectionID: R. Pooma 91033; institutionCode: GH
- **Type status:** Other material. **Occurrence:** recordedBy: Y. Ito; **Location:** country: Thailand; locality: Chiang Mai; Mae Sanam Mai village, Baw Sahlee subdistrict; verbatimLatitude: 18° 58' 44" N; verbatimLongitude: 98° 56' 3" E; **Event:** eventDate: Jul. 2, 2000; **Record Level:** collectionID: J.F. Maxwell 00-307; institutionCode: GH
- **Type status:** Other material. **Occurrence:** recordedBy: Y. Ito; **Location:** country: Thailand; locality: Chiang Mai; Mae Ria District, Doi Suthep-Pui National Park, N side; verbatimLatitude: 18° 49' 28" N; verbatimLongitude: 98° 53' 24" E; **Event:** eventDate: Feb. 20, 1990; **Record Level:** collectionID: J.F. Maxwell 90-216; institutionCode: GH
- **Type status:** Other material. **Occurrence:** recordedBy: Y. Ito; **Location:** country: Myanmar; locality: Shan State; Taungyi, Namkok; **Event:** eventDate: May. 1, 1938; **Record Level:** collectionID: F. G. Dickason 9405; institutionCode: GH
- **Type status:** Other material. **Occurrence:** recordedBy: Y. Ito; **Location:** country: Myanmar; locality: Shan State; Inle lake; **Event:** eventDate: May. 1, 1938; **Record Level:** collectionID: F. G. Dickason 7865; institutionCode: GH
- **Type status:** Other material. **Occurrence:** recordedBy: Y. Ito; **Location:** country: Myanmar; locality: Shan State, Kalow; **Event:** eventDate: Nov. 26, 2008; **Record Level:** collectionID: Tanaka et al. 080046; institutionCode: TI
- **Type status:** Other material. **Occurrence:** recordedBy: Y. Ito; **Location:** country: Myanmar; locality: Shan State, Pindaya; verbatimLatitude: 20° 59' 57'' N; verbatimLongitude: 96° 39' 59'' E; **Event:** eventDate: Dec. 1, 2008; **Record Level:** collectionID: Tanaka et al. 080623; institutionCode: TI
- **Type status:** Other material. **Occurrence:** recordedBy: Y. Ito; **Location:** country: Thailand; locality: Chiang Mai; Mai Muang Nao Arboretum, Bam Mae Sanam Mai, Baw Salee Subdistrict; verbatimLatitude: 18° 9' 41" N; verbatimLongitude: 98° 16' 54" E; **Event:** eventDate: Apr. 11, 2001; **Record Level:** collectionID: W. Sankamethawee 150; institutionCode: GH
- **Type status:** Other material. **Occurrence:** recordedBy: Y. Ito; **Location:** country: Thailand; locality: Chiang Mai Province; Hot District; Maesanaam Pine Inprovement Center; verbatimLatitude: 18° 11' N; verbatimLongitude: 98° 36' E; **Event:** eventDate: Jul. 8, 1995; **Record Level:** collectionID: R. Pooma et al. 1032; institutionCode: BKF
- **Type status:** Other material. **Occurrence:** recordedBy: Y. Ito; **Location:** country: Thailand; locality: Chiang Mai Province; Om-koi; **Event:** eventDate: Jul. 20, 1987; **Record Level:** collectionID: C. Phengklai et al. 6277; institutionCode: BKF

##### Distribution

Bangladesh, Bhutan, China (nationwide), India (nationwide), Indonesia (Borneo, Java, Sulawesi), Japan, Malaysia (Peninsular), Myanmar, Nepal, Pakistan, Philippines, Thailand; Oceania.

#### Hydrocharitaceae

#### Blyxa

Noronha ex Thouars, 1806

#### Blyxa aubertii

Rich., 1812

##### Materials

- **Type status:** Other material. **Occurrence:** recordedBy: Y. Ito; **Location:** country: Myanmar; verbatimLatitude: 16° 53' 19" N; verbatimLongitude: 95° 52' 29" E; **Record Level:** collectionID: MBK041203; institutionCode: TI
- **Type status:** Other material. **Occurrence:** recordedBy: Y. Ito; **Location:** country: Thailand; locality: Trat Province; 20 km W of Trat; verbatimLatitude: 12° 30' N; verbatimLongitude: 102° 20' E; **Event:** eventDate: Aug. 30, 1972; **Record Level:** collectionID: K. Larsen et al. 32232; institutionCode: AAU
- **Type status:** Other material. **Occurrence:** recordedBy: Y. Ito; **Location:** country: Thailand; locality: Trat Province; 18 km W of Trat; verbatimLatitude: 12° 40' N; verbatimLongitude: 102° 16' E; **Event:** eventDate: Oct. 23, 1972; **Record Level:** collectionID: J.F. Maxwell 72-540; institutionCode: AAU
- **Type status:** Other material. **Occurrence:** recordedBy: Y. Ito; **Location:** country: Thailand; locality: Chantaburi province; Plain of Makam; verbatimLatitude: 12° 41' N; verbatimLongitude: 102° 15' E; **Event:** eventDate: Aug. 22, 1966; **Record Level:** collectionID: K. Larsen et al. 1667; institutionCode: AAU
- **Type status:** Other material. **Occurrence:** recordedBy: Y. Ito; **Location:** country: Thailand; locality: Rayong Province; Ban phe; verbatimLatitude: 12° 40' N; verbatimLongitude: 101° 25' E; **Event:** eventDate: Dec. 16, 1974; **Record Level:** collectionID: R. Geesik & P. Hiepko 7872; institutionCode: AAU
- **Type status:** Other material. **Occurrence:** recordedBy: Y. Ito; **Location:** country: Thailand; locality: Chantaburi Province; S of Mekam rubber plantation; verbatimLatitude: 12° 35' N; verbatimLongitude: 102° 20' E; **Event:** eventDate: Aug. 22, 1966; **Record Level:** collectionID: K. Larsen et al. 1744; institutionCode: AAU
- **Type status:** Other material. **Occurrence:** recordedBy: Y. Ito; **Location:** country: Thailand; locality: Narathiwat Province; Tak bai; verbatimLatitude: 6° 15' 34" N; verbatimLongitude: 102° 0' 16" E; **Event:** eventDate: Jan. 11, 1986; **Record Level:** collectionID: C. Cniyomdham 1117; institutionCode: AAU
- **Type status:** Other material. **Occurrence:** recordedBy: Y. Ito; **Location:** country: Thailand; locality: Phangnga Province; Takuapah District; 15 km N of Takuapah; verbatimLatitude: 8° 53 'N; verbatimLongitude: 98° 21' E; **Event:** eventDate: Jul. 14, 1972; **Record Level:** collectionID: K. Larsen et al. 30970; institutionCode: AAU
- **Type status:** Other material. **Occurrence:** recordedBy: Y. Ito; **Location:** country: Vietnam; locality: Lao Cai Province; Than Uyen District, Municipality Ho Mit; verbatimLatitude: 22° 6' N; verbatimLongitude: 103° 52' E; **Event:** eventDate: May. 21, 1999; **Record Level:** collectionID: NTH2708; institutionCode: AAU

##### Distribution

Bangladesh, China (Southern), India (Western, Central, Southern), Indonesia (Java, Sumatra), Japan, Malaysia (Peninsular), Myanmar, ?Nepal, Papua New guinea, Sri Lanka, Thailand, Vietnam.

#### Blyxa echinosperma

(C.B. Clarke) Hook. f., 1888

##### Materials

- **Type status:** Other material. **Occurrence:** recordedBy: Y. Ito; **Location:** country: Myanmar; locality: Kachin State; verbatimLatitude: 26° 40' 50'' N; verbatimLongitude: 96° 15' 20'' E; **Event:** eventDate: Dec. 10, 2005; **Record Level:** collectionID: J. Murata et al. 040940; institutionCode: TI
- **Type status:** Other material. **Occurrence:** recordedBy: Y. Ito; **Location:** country: Thailand; locality: Phangnga Province, Khuraburi, Ko Phrathong; verbatimLatitude: 9° 11' 45" N; verbatimLongitude: 98° 24' 30" E; **Event:** eventDate: Aug. 24, 2005; **Record Level:** collectionID: C. Phengklai et al. 15062; institutionCode: BKF
- **Type status:** Other material. **Occurrence:** recordedBy: Y. Ito; **Location:** country: Thailand; locality: Chiang Mai; near Angka Noi village, N side of the main road, Doi Inthanon.; verbatimLatitude: 18° 35' 32" N; verbatimLongitude: 98° 29' 12" E; **Event:** eventDate: May. 8, 1988; **Record Level:** collectionID: N. Fukuoka T-62538; institutionCode: TI

##### Distribution

Bangladesh, China (Central, Southern), India (Western, Central, Southern), Indonesia (Java), Japan, Malaysia (Borneo, Peninsular), Myanmar, ?Nepal, Papua New Guinea, Philippines, Thailand, Sri Lanka; Oceania.

#### Blyxa japonica

(Miq.) Maxim. ex Asch. & Gürke, 1889

##### Materials

- **Type status:** Other material. **Occurrence:** recordedBy: Y. Ito; **Location:** country: Cambodia; locality: Kampot Province; Kampot District, S summit of Phnom Bokor, Bokor village; verbatimLatitude: 10° 37' 4" N; verbatimLongitude: 104° 1' 41" E; **Event:** eventDate: Jun. 18, 1997; **Record Level:** collectionID: McDonald et al. 5793; institutionCode: AAU
- **Type status:** Other material. **Occurrence:** recordedBy: Y. Ito; **Location:** country: Thailand; locality: Naratiwat Province; Sungeipadi District; verbatimLatitude: 6° 29' 4" N; verbatimLongitude: 101° 49' 25" E; **Event:** eventDate: Mar. 28, 1987; **Record Level:** collectionID: J.F. Maxwell 87-265; institutionCode: GH
- **Type status:** Other material. **Occurrence:** recordedBy: Y. Ito; **Location:** country: Thailand; locality: E Si Saket Province; between Uthum Phon Phisai and Si Saket; verbatimLatitude: 15° 7' N; verbatimLongitude: 104° 15' E; **Event:** eventDate: Oct. 7, 1984; **Record Level:** collectionID: G. Murata, C. Phengklai, S. Mitsuta, H. Nagamasu, N. Nantasan T-49920; institutionCode: GH
- **Type status:** Other material. **Occurrence:** recordedBy: Y. Ito; **Location:** country: Thailand; locality: Kanchanaburi Province; Thong Pha Phum District, Rintin Forest; verbatimLatitude: 14° 50' 9" N; verbatimLongitude: 98° 44' 21" E; **Event:** eventDate: Nov. 6, 1979; **Record Level:** collectionID: T. Shimizu et al. T-21941; institutionCode: AAU
- **Type status:** Other material. **Occurrence:** recordedBy: Y. Ito; **Location:** country: Thailand; locality: Trat Province; 20 km W of Trat; verbatimLatitude: 12° 30' N; verbatimLongitude: 102° 20' E; **Event:** eventDate: Aug. 30, 1972; **Record Level:** collectionID: K. Larsen et al. 32234; institutionCode: AAU
- **Type status:** Other material. **Occurrence:** recordedBy: Y. Ito; **Location:** country: Thailand; locality: Nakhon Ratchasima Province; Khao yai Natl Park, Khao Laem; verbatimLatitude: 14° 45' N; verbatimLongitude: 102° E; **Event:** eventDate: Oct. 19, 1969; **Record Level:** collectionID: C.F. van Beusekom, C. Phengkhlai 1769; institutionCode: AAU
- **Type status:** Other material. **Occurrence:** recordedBy: Y. Ito; **Location:** country: Thailand; locality: Narathiwat Province; Tak bai; verbatimLatitude: 6° 14' 57" N; verbatimLongitude: 102° 0' 6" E; **Event:** eventDate: Jan. 11, 1986; **Record Level:** collectionID: C. Cniyomdham 1116; institutionCode: AAU
- **Type status:** Other material. **Occurrence:** recordedBy: Y. Ito; **Location:** country: Thailand; locality: Chantaburi Province; verbatimLatitude: 12° 57' N; verbatimLongitude: 102° E; **Event:** eventDate: Oct. 17, 1971; **Record Level:** collectionID: J.F. Maxwell 71-576; institutionCode: AAU
- **Type status:** Other material. **Occurrence:** recordedBy: Y. Ito; **Location:** country: Thailand; locality: Saraburi Province; Salum Lake; verbatimLatitude: 14° 32' N; verbatimLongitude: 101° 2' E; **Event:** eventDate: Oct. 14, 1973; **Record Level:** collectionID: J.F. Maxwell 73-504; institutionCode: AAU
- **Type status:** Other material. **Occurrence:** recordedBy: Y. Ito; **Location:** country: Thailand; locality: Pha Team National Park, Ubon Ratchathani; verbatimLatitude: 15° 24' 10'' N; verbatimLongitude: 105° 29' 23'' E; **Record Level:** collectionID: Nr. Tanaka; institutionCode: TNS

##### Distribution

Bangladesh, China (nationwide), Cambodia, India (Western [Meghalaya]), Indonesia (Boreo, New Guinea, Sulawesi), Japan, Malaysia (Peninsular), Myanmar, Papua New Guinea, Thailand.

#### Blyxa quadricostata

den Hartog, 1973

##### Materials

- **Type status:** Other material. **Occurrence:** recordedBy: Y. Ito; **Location:** country: Thailand; locality: Naratiwat Province; Sungei Padi District, Sungei Padi; verbatimLatitude: 6° 7' 27" N; verbatimLongitude: 101° 54' 47" E; **Event:** eventDate: Mar. 28, 1987; **Record Level:** collectionID: J.F. Maxwell 87-267; institutionCode: GH
- **Type status:** Other material. **Occurrence:** recordedBy: Y. Ito; **Location:** country: Thailand; locality: Loei Province; Phu Kradung; verbatimLatitude: 17° 16' 54" N; verbatimLongitude: 101° 9' 31" E; **Event:** eventDate: Nov. 29, 1965; **Record Level:** collectionID: M. Tagawa et al. T-781; institutionCode: AAU
- **Type status:** Other material. **Occurrence:** recordedBy: Y. Ito; **Location:** country: Thailand; locality: Loei Province; Phu Kradung; verbatimLatitude: 16° 53' N; verbatimLongitude: 101° 47' E; **Event:** eventDate: Nov. 2, 1984; **Record Level:** collectionID: G. Murata et al. T-42713; institutionCode: AAU
- **Type status:** Other material. **Occurrence:** recordedBy: Y. Ito; **Location:** country: Thailand; locality: Loei Province; Phu Kradung District, Phu Kradung Natl park; verbatimLatitude: 17° 16' 54" N; verbatimLongitude: 101° 9' 31" E; **Event:** eventDate: Nov. 15, 1979; **Record Level:** collectionID: T. Shimizu et al. T-22824; institutionCode: AAU
- **Type status:** Other material. **Occurrence:** recordedBy: Y. Ito; **Location:** country: Thailand; locality: Loei Province; Phu Kradung District, Phu Kradung Natl park; verbatimLatitude: 17° 16' 54" N; verbatimLongitude: 101° 9' 31" E; **Event:** eventDate: Mar. 9, 1979; **Record Level:** collectionID: P.J. O'Connor & C. Niyondham 15711; institutionCode: AAU
- **Type status:** Other material. **Occurrence:** recordedBy: Y. Ito; **Location:** country: Thailand; locality: Loei Province; Phu Kradung; verbatimLatitude: 17° 17' N; verbatimLongitude: 101° 10' E; **Event:** eventDate: Mar. 18, 1958; **Record Level:** collectionID: Th. Sorensen 2211; institutionCode: AAU
- **Type status:** Other material. **Occurrence:** recordedBy: Y. Ito; **Location:** country: Thailand; locality: Loei Province; Phu Kradung, S. of Loi; verbatimLatitude: 16° 53' N; verbatimLongitude: 101° 53' E; **Event:** eventDate: Nov. 7, 1970; **Record Level:** collectionID: Ch. Charoenphol et al. 4783; institutionCode: AAU
- **Type status:** Holotype. **Occurrence:** recordedBy: Y. Ito; **Location:** country: Thailand; locality: Loei Province; Phu Kradung, Mie ron Pond; verbatimLatitude: 16° 52' N; verbatimLongitude: 101° 52' E; **Event:** eventDate: Dec. 25, 1971; **Record Level:** collectionID: C.F. van Beusekom et al. 4602; institutionCode: MO

##### Distribution

Thailand.

#### Egeria

Planch., 1849

#### Egeria densa

(Planch.) Casp., 1857

##### Materials

- **Type status:** Other material. **Occurrence:** recordedBy: Y. Ito; **Location:** country: Thailand; locality: Bung Bonapet Nonhunting Area; verbatimLatitude: 15° 41' 40" N; verbatimLongitude: 100° 16' 03" E; **Event:** eventDate: Nov. 16, 2012; **Record Level:** collectionID: Y. Ito 1733; institutionCode: BKF

##### Distribution

Native to South America.

#### Elodea

Michx., 1803

#### Elodea nuttallii

(Planch.) H. St. John, 1920

##### Distribution

Native to North America.

#### Hydrilla

Rich., 1814

#### Hydrilla verticillata

(L. f.) Royle, 1839

##### Materials

- **Type status:** Other material. **Occurrence:** recordedBy: Y. Ito; **Location:** country: Myanmar; stateProvince: Kachin; verbatimLocality: Tanaing Township; verbatimLatitude: 26° 22' 22'' N; verbatimLongitude: 96° 43' E; **Event:** eventDate: Sep. 15, 2005; **Record Level:** collectionID: MBK040056; institutionCode: TI
- **Type status:** Other material. **Occurrence:** recordedBy: Y. Ito; **Location:** country: Myanmar; stateProvince: Shan State; locality: Pindaya; verbatimLatitude: 20° 59' 57" N; verbatimLongitude: 96° 39' 59" E; **Record Level:** collectionID: MBK080634; institutionCode: TI
- **Type status:** Other material. **Occurrence:** recordedBy: Y. Ito; **Location:** country: Myanmar; locality: Mandalay Division; verbatimLatitude: 22° 00' 29'' N; verbatimLongitude: 96° 28' 06'' E; **Event:** eventDate: Jan. 11, 2002; **Record Level:** collectionID: Tanaka et al. 021712; institutionCode: TI
- **Type status:** Other material. **Occurrence:** recordedBy: Y. Ito; **Location:** country: Myanmar; locality: Mandalay Division; verbatimLatitude: 20° 48' N; verbatimLongitude: 95° 15' E; **Event:** eventDate: Mar. 4, 2003; **Record Level:** collectionID: Tanaka et al. 028704; institutionCode: TI
- **Type status:** Other material. **Occurrence:** recordedBy: Y. Ito; **Location:** country: Myanmar; locality: Bago Division; verbatimLatitude: 18° 42' 22'' N; verbatimLongitude: 95° 5' 59'' E; **Event:** eventDate: Dec. 9, 2006; **Record Level:** collectionID: Sugawara et al. 036433; institutionCode: TI
- **Type status:** Other material. **Occurrence:** recordedBy: Y. Ito; **Location:** country: Myanmar; locality: Kachin State; verbatimLatitude: 26° 6' 34'' N; verbatimLongitude: 96° 42' 58'' E; **Event:** eventDate: Sep. 19, 2005; **Record Level:** collectionID: Tanaka et al. 040483; institutionCode: TI
- **Type status:** Other material. **Occurrence:** recordedBy: Y. Ito; **Location:** country: Myanmar; locality: Shan State; verbatimLatitude: 20° 35' 41'' N; verbatimLongitude: 96° 31' 46'' E; **Event:** eventDate: Nov. 26, 2008; **Record Level:** collectionID: Tanaka et al. 080050; institutionCode: TI
- **Type status:** Other material. **Occurrence:** recordedBy: Y. Ito; **Location:** country: Myanmar; locality: Shan State; verbatimLatitude: 20° 35' 41'' N; verbatimLongitude: 96° 31' 46'' E; **Event:** eventDate: Nov. 26, 2008; **Record Level:** collectionID: Tanaka et al. 080057; institutionCode: TI
- **Type status:** Other material. **Occurrence:** recordedBy: Y. Ito; **Location:** country: Myanmar; locality: Shan State; verbatimLatitude: 20° 27' 28'' N; verbatimLongitude: 96° 50' 37'' E; **Event:** eventDate: Dec. 4, 2008; **Record Level:** collectionID: Tanaka et al. 080663; institutionCode: TI
- **Type status:** Other material. **Occurrence:** recordedBy: Y. Ito; **Location:** country: Myanmar; locality: Taunngyi; verbatimLatitude: 20° 47' 14" N; verbatimLongitude: 97° 2' 7" E; **Event:** eventDate: Jan. 5, 1938; **Record Level:** collectionID: F.G. Dickason 9334; institutionCode: GH
- **Type status:** Other material. **Occurrence:** recordedBy: Y. Ito; **Location:** country: Thailand; locality: Chiang Mai Province; Chiang Dao District; verbatimLatitude: 19° 28' 46" N; verbatimLongitude: 98° 54' 44" E; **Event:** eventDate: Dec. 11, 1992; **Record Level:** collectionID: V.A. Sunthorn, P. Palee 101; institutionCode: GH
- **Type status:** Other material. **Occurrence:** recordedBy: Y. Ito; **Location:** country: Thailand; verbatimLatitude: 13° 45' 10" N; verbatimLongitude: 100° 29' 45" E; **Record Level:** collectionID: T- 50413 [TI]; institutionCode: TI
- **Type status:** Other material. **Occurrence:** recordedBy: Y. Ito; **Location:** country: Thailand; locality: Pha Team National Park, Ubon Ratchathani, Thailand; verbatimLatitude: 15° 24' 07'' N; verbatimLongitude: 105° 29' 20'' E; **Record Level:** collectionID: Tr. Tanaka; institutionCode: TNS
- **Type status:** Other material. **Occurrence:** recordedBy: Y. Ito; **Location:** country: Thailand; locality: Hotel river Kwai, Kantchanabury; verbatimLatitude: 14° 1' 59" N; verbatimLongitude: 99° 31' 10" E; **Event:** eventDate: Nov. 15, 2012; **Record Level:** collectionID: Y. Ito 1724; institutionCode: BKF

##### Distribution

Bangladesh, Bhutan, China (nationwide), Indonesia (nationwide), Japan, Malaysia (nationwide), Myanmar, Nepal, Pakistan, Papua New Guinea, Thailand, Sri Lanka.

#### Hydrocharis

L., 1753

#### Hydrocharis dubia

(Blume) Backer, 1925

##### Materials

- **Type status:** Other material. **Occurrence:** recordedBy: Y. Ito; **Location:** country: Thailand; locality: Ang Thong Province; Howa Pie; verbatimLatitude: 13° 54' N; verbatimLongitude: 100° 37' E; **Event:** eventDate: Sep. 17, 1972; **Record Level:** collectionID: J.F. Maxwell 72-398; institutionCode: AAU
- **Type status:** Other material. **Occurrence:** recordedBy: Y. Ito; **Location:** country: Thailand; locality: Saraburi Province; Muang District, Sahm Lahn forest; verbatimLatitude: 14° 31' 51" N; verbatimLongitude: 100° 54' 34" E; **Event:** eventDate: Oct. 20, 1974; **Record Level:** collectionID: J.F. Maxwell 74-947; institutionCode: AAU
- **Type status:** Other material. **Occurrence:** recordedBy: Y. Ito; **Location:** country: Thailand; verbatimLatitude: 13° 45' 10" N; verbatimLongitude: 100° 29' 45" E; **Record Level:** collectionID: 16322; institutionCode: BKF
- **Type status:** Other material. **Occurrence:** recordedBy: Y. Ito; **Location:** country: Thailand; locality: Maha Sarakham; Bua Khaaw Morning Market; verbatimLatitude: 16° 11' N; verbatimLongitude: 103° 18' E; **Event:** eventDate: Mar. 11, 1990; **Record Level:** collectionID: Mooly Widmer 0699; institutionCode: BKF

##### Distribution

China (nationwide), Indonesia (Java, New Guinea, Sulawesi), Japan, ?Myanmar, Papua New Guinea, Philippines, Thailand, ?Vietnam; Oceania.

#### Najas

L., 1753

#### Najas graminea

Delile, 1813

##### Materials

- **Type status:** Other material. **Occurrence:** recordedBy: Y. Ito; **Location:** country: Myanmar; locality: Shan State, Inle Lake; verbatimLatitude: 20° 32' 2'' N; verbatimLongitude: 96° 53' 53'' E; **Event:** eventDate: Dec. 3, 2008; **Record Level:** collectionID: Tanaka et al. 080656; institutionCode: MBK
- **Type status:** Other material. **Occurrence:** recordedBy: Y. Ito; **Location:** country: Thailand; locality: Mae Hong Son Province; verbatimLatitude: 19° 15' N; verbatimLongitude: 98° E; **Event:** eventDate: Sep. 10, 1974; **Record Level:** collectionID: K. Larsen & S.S. Larsen KL34376; institutionCode: BKF
- **Type status:** Other material. **Occurrence:** recordedBy: Y. Ito; **Location:** country: Thailand; locality: Pangnga Province; Kan bow koranee cascade; verbatimLatitude: 8° 25' N; verbatimLongitude: 98° 30' E; **Event:** eventDate: May. 9, 1973; **Record Level:** collectionID: R. Geesink & T. Santisuk 5299; institutionCode: AAU
- **Type status:** Other material. **Occurrence:** recordedBy: Y. Ito; **Location:** country: Thailand; locality: Petchabury; verbatimLatitude: 13° 30' 6" N; verbatimLongitude: 99° 47' 37" E; **Event:** eventDate: Nov. 14, 2012; **Record Level:** collectionID: Y. Ito 1714; institutionCode: BKF

##### Distribution

Bangladesh, China (Central, Southern), India (Central, Southern), Indonesia (Java, New Guinea, Sumatra, Sulawesi), Japan, Myanmar, Nepal, Papua New Guinea, Philippines, Thailand, ?Vietnam; Africa; Oceania.

#### Najas indica

(Willd.) Cham., 1829

##### Materials

- **Type status:** Other material. **Occurrence:** recordedBy: Y. Ito; **Location:** country: Myanmar; verbatimLatitude: 16° 53' 19.18"; verbatimLongitude: 95° 52' 28.59"; **Record Level:** collectionID: MBK020915; institutionCode: TI
- **Type status:** Other material. **Occurrence:** recordedBy: Y. Ito; **Location:** country: Myanmar; verbatimLatitude: 16° 53' 19.18"; verbatimLongitude: 95° 52' 28.59"; **Record Level:** collectionID: MBK036320; institutionCode: TI
- **Type status:** Other material. **Occurrence:** recordedBy: Y. Ito; **Location:** country: Thailand; locality: Ubon Ratchathani Province; Kang Tana Natl park; verbatimLatitude: 15° 16' 26" N; verbatimLongitude: 105° 27' 31" E; **Event:** eventDate: Aug. 22, 2001; **Record Level:** collectionID: R. Pooma et al. 2346; institutionCode: BKF
- **Type status:** Other material. **Occurrence:** recordedBy: Y. Ito; **Location:** country: Thailand; locality: Chaiyaphum Province; Tunhamang; verbatimLatitude: 16° 20' N; verbatimLongitude: 101° 45' E; **Event:** eventDate: Dec. 19, 1971; **Record Level:** collectionID: van Beusekom et al. 4245; institutionCode: BKF
- **Type status:** Other material. **Occurrence:** recordedBy: Y. Ito; **Location:** country: Thailand; locality: Petchabury; verbatimLatitude: 13° 30' 6" N; verbatimLongitude: 99° 47' 37" E; **Event:** eventDate: Nov. 14, 2012; **Record Level:** collectionID: Y. Ito 1713; institutionCode: BKF

##### Distribution

Bangladesh, India (Western, Central, Southern), Indonesia (Java, Sumatra, Sulawesi), Myanmar, Papua New Guinea, Sri Lanka, Thailand, Vietnam.

#### Najas marina

L., 1753

##### Materials

- **Type status:** Other material. **Occurrence:** recordedBy: Y. Ito; **Location:** country: Thailand; locality: Phatthalung Province; Thale noi Bord reservior; verbatimLatitude: 7° 44' N; verbatimLongitude: 100° 9' E; **Event:** eventDate: Oct. 7, 2005; **Record Level:** collectionID: H.-J. Esser et al. 05-91; institutionCode: BKF
- **Type status:** Other material. **Occurrence:** recordedBy: Y. Ito; **Location:** country: Thailand; locality: Phatthalung Province; Sea Thale Noi; verbatimLatitude: 7° 47' 9" N; verbatimLongitude: 100° 10' 4" E; **Event:** eventDate: Oct. 7, 2005; **Record Level:** collectionID: Tillich et al. 5094; institutionCode: BKF
- **Type status:** Other material. **Occurrence:** recordedBy: Y. Ito; **Location:** country: Thailand; locality: Khao San Yot Natl Park.; verbatimLatitude: 12° 14' 39" N; verbatimLongitude: 99° 55' 57" E; **Event:** eventDate: Nov. 13, 2012; **Record Level:** collectionID: Y. Ito 1701; institutionCode: BKF

##### Distribution

Worldwide.

#### Najas tenuis

Magnus, 1870

##### Materials

- **Type status:** Other material. **Occurrence:** recordedBy: Y. Ito; **Location:** country: Myanmar; locality: Shan State; Naungkhio, Hsipaw Distr.; verbatimLatitude: 22° 37' 12" N; verbatimLongitude: 97° 17' E; **Event:** eventDate: Oct. 1, 1939; **Record Level:** collectionID: F. G. Dickason 9565; institutionCode: GH
- **Type status:** Other material. **Occurrence:** recordedBy: Y. Ito; **Location:** country: Myanmar; locality: Shan State, Inle Lake; verbatimLatitude: 20° 32' 2'' N; verbatimLongitude: 96° 53' 53'' E; **Event:** eventDate: Dec. 3, 2008; **Record Level:** collectionID: Tanaka et al. 080642; institutionCode: TI

##### Distribution

India, Myanmar.

##### Notes

Fig. 3.

#### Nechamandra

Planch., 1849

#### Nechamandra alternifolia

(Roxb.) Thwaites, 1864

##### Materials

- **Type status:** Other material. **Occurrence:** recordedBy: Y. Ito; **Location:** country: Myanmar; locality: Pindaya, Shan State; verbatimLatitude: 20° 59' 57'' N; verbatimLongitude: 96° 39' 59'' E; **Event:** eventDate: Dec. 1, 2008; **Record Level:** collectionID: Tanaka et al. 080635; institutionCode: MBK
- **Type status:** Other material. **Occurrence:** recordedBy: Y. Ito; **Location:** country: Myanmar; locality: Kalow, Shan State; verbatimLatitude: 20° 35' 41'' N; verbatimLongitude: 96° 31' 46'' E; **Event:** eventDate: Nov. 26, 2008; **Record Level:** collectionID: Tanaka et al. 080053; institutionCode: TI
- **Type status:** Other material. **Occurrence:** recordedBy: Y. Ito; **Location:** country: Myanmar; locality: Kalow, Shan State; verbatimLatitude: 20° 35' 41'' N; verbatimLongitude: 96° 31' 46'' E; **Event:** eventDate: Nov. 26, 2008; **Record Level:** collectionID: Tanaka et al. 080058; institutionCode: TI
- **Type status:** Other material. **Occurrence:** recordedBy: Y. Ito; **Location:** country: Myanmar; locality: Kalow, Shan State; verbatimLatitude: 20° 35' 41'' N; verbatimLongitude: 96° 31' 46'' E; **Event:** eventDate: Nov. 26, 2008; **Record Level:** collectionID: Tanaka et al. 080059; institutionCode: TI
- **Type status:** Other material. **Occurrence:** recordedBy: Y. Ito; **Location:** country: Thailand; locality: Pathum Thani Province; along way to the young delta; verbatimLatitude: 14° 1' 34" N; verbatimLongitude: 100° 31' 29" E; **Event:** eventDate: Jul. 30, 1973; **Record Level:** collectionID: G. Murata & N. Fukuoka T-17324; institutionCode: BKF

##### Distribution

Bangladesh, China (Southern), India (Eastern, Northern, Southern), Myanmar, Nepal, Sri Lanka, Thailand, Vietnam; Yemen, and Sudan.

##### Notes

Fig. 4.

Myanmar (Ito et al. 2009); Thailand (Ito 2013b).

#### Ottelia

Pers., 1805

#### Ottelia alismoides

(L.) Pers., 1805

##### Materials

- **Type status:** Other material. **Occurrence:** recordedBy: Y. Ito; **Location:** country: Laos; locality: Vientine; verbatimLatitude: 17° 58' N; verbatimLongitude: 102° 36' E; **Event:** eventDate: Dec. 18, 1957; **Record Level:** collectionID: T. Tsuyama L. 57001; institutionCode: TI
- **Type status:** Other material. **Occurrence:** recordedBy: Y. Ito; **Location:** country: Myanmar; locality: Mandaley; verbatimLatitude: 21° 58' 30" N; verbatimLongitude: 96° 5' E; **Event:** eventDate: Jan. 14, 1964; **Record Level:** collectionID: H. Kanai; institutionCode: TI
- **Type status:** Other material. **Occurrence:** recordedBy: Y. Ito; **Location:** country: Myanmar; locality: Kachin State; verbatimLatitude: 26° 6' 34'' N; verbatimLongitude: 96° 42' 58'' E; **Event:** eventDate: Sep. 19, 2005; **Record Level:** collectionID: TI040482; institutionCode: TI
- **Type status:** Other material. **Occurrence:** recordedBy: Y. Ito; **Location:** country: Myanmar; locality: Kachin State; at the evening market of Tanaing, Hukaung Valley, Tanaing Township; verbatimLatitude: 26° 31' 20" N; verbatimLongitude: 96° 35' 9" E; **Event:** eventDate: Sep. 20, 2005; **Record Level:** collectionID: TI040488; institutionCode: TI
- **Type status:** Other material. **Occurrence:** recordedBy: Y. Ito; **Location:** country: Myanmar; locality: Mandalay Division; between Smithine town and Pinchar village, ca. 25 miles S of Mandalay; verbatimLatitude: 21° 29' 47" N; verbatimLongitude: 96° 4' 21" E; **Event:** eventDate: Jun. 19, 2000; **Record Level:** collectionID: TI2026; institutionCode: TI
- **Type status:** Other material. **Occurrence:** recordedBy: Y. Ito; **Location:** country: Thailand; locality: Chokchai, Nakhon Ratchasima, Thailand; verbatimLatitude: 14° 44' 30'' N; verbatimLongitude: 102° 9' 41'' E; **Record Level:** collectionID: Nr. Tanaka; institutionCode: TNS
- **Type status:** Other material. **Occurrence:** recordedBy: Y. Ito; **Location:** country: Thailand; locality: Petchabury; verbatimLatitude: 13° 24' 30" N; verbatimLongitude: 99° 48' 44" E; **Event:** eventDate: Nov. 14, 2012; **Record Level:** collectionID: Y. Ito 1707; institutionCode: BKF
- **Type status:** Other material. **Occurrence:** recordedBy: Y. Ito; **Location:** country: Thailand; locality: Khuen Shinaga Natl Park.; verbatimLatitude: 14° 38' 8" N; verbatimLongitude: 98° 59' 52" E; **Event:** eventDate: Nov. 15, 2012; **Record Level:** collectionID: Y. Ito 1726; institutionCode: BKF
- **Type status:** Other material. **Occurrence:** recordedBy: Y. Ito; **Location:** country: Thailand; locality: Kantchanabury; verbatimLatitude: 14° 39' 21" N; verbatimLongitude: 98° 42' 27" E; **Event:** eventDate: Nov. 15, 2012; **Record Level:** collectionID: Y. Ito 1731; institutionCode: BKF
- **Type status:** Other material. **Occurrence:** recordedBy: Y. Ito; **Location:** country: Thailand; locality: Nongkhai Province; Bungkhla District, Ban Dong Mak Yang; verbatimLatitude: 18° 19' 59'' N; verbatimLongitude: 103° 43' 39'' E; **Event:** eventDate: Aug. 27, 2001; **Record Level:** collectionID: R. Pooma, W.J.J.O. de Wilde, B.E.E. Duyfjes, V. Chamchumroon, K. Phattarahirankanok 2864; institutionCode: GH
- **Type status:** Other material. **Occurrence:** recordedBy: Y. Ito; **Location:** country: Thailand; locality: Kanchanaburi Province; Thong Pha Phum District, Rintin Forest; verbatimLatitude: 14° 48' 34" N; verbatimLongitude: 98° 44' 41" E; **Event:** eventDate: Nov. 6, 1979; **Record Level:** collectionID: T. Simizu, H. Toyokuni, H. Koyama, T. Yahara, C. Niyomdham T-21923; institutionCode: GH
- **Type status:** Other material. **Occurrence:** recordedBy: Y. Ito; **Location:** country: Thailand; locality: Uthaithani Province; Larn Suk District, Huay Kha Kaeng Wildlife Sanctuary; verbatimLatitude: 15° 24' 30" N; verbatimLongitude: 100° 4' 35" E; **Event:** eventDate: Nov. 11, 1979; **Record Level:** collectionID: T. Simizu, H. Toyokuni, H. Koyama, T. Yahara, C. Niyomdham T-22221; institutionCode: GH
- **Type status:** Other material. **Occurrence:** recordedBy: Y. Ito; **Location:** country: Thailand; locality: Chainart Province; verbatimLatitude: 15° 14' N; verbatimLongitude: 100° 15' E; **Event:** eventDate: Nov. 28, 1959; **Record Level:** collectionID: L.B. & E.C. Abbe, T. Smitinand, B. Rollet 9233; institutionCode: GH
- **Type status:** Other material. **Occurrence:** recordedBy: Y. Ito; **Location:** country: Thailand; locality: Songkla Province; Haad Yai District, ban Koke Saht, W of Toong Loong; verbatimLatitude: 7° 0' 13" N; verbatimLongitude: 100° 27' 25" E; **Event:** eventDate: Oct. 2, 1985; **Record Level:** collectionID: J.F. Maxwell 85-929; institutionCode: GH
- **Type status:** Other material. **Occurrence:** recordedBy: Y. Ito; **Location:** country: Thailand; locality: Hue san - Chieing Rae; verbatimLatitude: 19° 54' 34" N; verbatimLongitude: 99° 49' 39" E; **Event:** eventDate: Nov. 21, 1921; **Record Level:** collectionID: B. Hayata; institutionCode: TI

##### Distribution

Cambodia, India, Indonesia, Japan, Korea, Laos, Malaysia, Myanmar, Nepal, New Guinea, Philippines, Thailand, Sri Lanka, Vietnam; Africa, Australia.

#### Ottelia cordata

(Wall.) Dandy, 1934

##### Materials

- **Type status:** Other material. **Occurrence:** recordedBy: Y. Ito; **Location:** country: Myanmar; locality: Mandalay Division; ca. 3 miles from Kyauk Sei, Pyin U Lwin (May Myo); verbatimLatitude: 21° 36' N; verbatimLongitude: 96° 4' 6" E; **Event:** eventDate: Mar. 16, 2003; **Record Level:** collectionID: N. Kuroiwa et al. 020926; institutionCode: TI

##### Distribution

?Cambodia, China (Southern [Hainan]), Myanmar, ?Thailand.

#### Vallisneria

L., 1753

#### Vallisneria spiralis

L., 1753

##### Materials

- **Type status:** Other material. **Occurrence:** recordedBy: Y. Ito; **Location:** country: Myanmar; locality: Shan State; Inle Lake; verbatimLatitude: 16° 53' 19" N; verbatimLongitude: 95° 52' 29" E; **Event:** eventDate: Dec. 3, 2008; **Record Level:** collectionID: Tanaka et al. 080653; institutionCode: MBK
- **Type status:** Other material. **Occurrence:** recordedBy: Y. Ito; **Location:** country: Myanmar; locality: Kachin State; between Khalone and Shinbweyan; verbatimLatitude: 26° 40' 50'' N; verbatimLongitude: 96° 15' 20'' E; **Event:** eventDate: Dec. 5, 2005; **Record Level:** collectionID: Murata et al.041208; institutionCode: TI
- **Type status:** Other material. **Occurrence:** recordedBy: Y. Ito; **Location:** country: Myanmar; locality: Kachin State; between Shinbweyan and Tanain; verbatimLatitude: 26° 56' N; verbatimLongitude: 96° 52' E; **Event:** eventDate: Feb. 16, 2007; **Record Level:** collectionID: Murata et al. 041622; institutionCode: TI

##### Distribution

Bangladesh, China, India (nationwide), Japan, Myanmar, Nepal, Pakistan, Sri Lanka.

#### Aponogetonaceae

#### Aponogeton

L. f., 1781

#### Aponogeton lakhonensis

A. Camus, 1910

##### Materials

- **Type status:** Other material. **Occurrence:** recordedBy: Y. Ito; **Location:** country: Myanmar; locality: Kachin State; between Takhet Village and Khalone Village, ca 10 mi E of Shinbwiyang; verbatimLatitude: 26° 38' 59" N; verbatimLongitude: 96° 20' 55" E; **Event:** eventDate: Dec. 10, 2005; **Record Level:** collectionID: Tanaka et al. 040939; institutionCode: TI
- **Type status:** Other material. **Occurrence:** recordedBy: Y. Ito; **Location:** country: Myanmar; locality: Kachin State; along the Ledo Rd between Shinbwiyang and Tanaing; verbatimLatitude: 26° 34' N; verbatimLongitude: 96° 30' E; **Event:** eventDate: Feb. 16, 2007; **Record Level:** collectionID: Tanaka et al. 041619; institutionCode: TI
- **Type status:** Other material. **Occurrence:** recordedBy: Y. Ito; **Location:** country: Thailand; locality: Lampang Province; verbatimLatitude: 18° 19' 24" N; verbatimLongitude: 99° 29' 29" E; **Event:** eventDate: Sep. 1, 1926; **Record Level:** collectionID: V.N. Pak Kuap 1797; institutionCode: BKF

##### Distribution

Cambodia, China (Southern), India (Eastern [Assam, Darjeeling]), Indonesia (Sulawesi), Myanmar, Thailand, Vietnam.

##### Notes

Fig. 5.

#### Potamogetonaceae

#### Potamogeton

L., 1753

#### Potamogeton crispus

L., 1753

##### Materials

- **Type status:** Other material. **Occurrence:** recordedBy: Y. Ito; **Location:** country: Thailand; locality: Sagaing Division; Kalewa-Kalemyo; verbatimLatitude: 23° 12' 17" N; verbatimLongitude: 94° 11' 33" E; **Event:** eventDate: Mar. 25, 1938; **Record Level:** collectionID: F. G. Dickason 7196; institutionCode: GH
- **Type status:** Other material. **Occurrence:** recordedBy: Y. Ito; **Location:** country: Myanmar; locality: Sagaing Division; Kalewa-Kalemyo, shallow water, Alt. 600 ft.; verbatimLatitude: 23° 12' 17" N; verbatimLongitude: 94° 11' 33" E; **Event:** eventDate: Mar. 26, 1938; **Record Level:** collectionID: F. G. Dickason 7204; institutionCode: GH

##### Distribution

Bangladesh, Bhutan, China (nationwide), India (nationwide), Japan, Korea, Laos, Malesia (Sumatra), Myanmar, Nepal, Pakistan, Vietnam; Worldwide.

#### Potamogeton distinctus

A. Benn., 1904

##### Materials

- **Type status:** Other material. **Occurrence:** recordedBy: Y. Ito; **Location:** country: Thailand; locality: Khuen Shinaga Natl Park; Nearn Saran View point.; verbatimLatitude: 14° 44' 25" N; verbatimLongitude: 98° 49' 6" E; **Event:** eventDate: Nov. 15, 2012; **Record Level:** collectionID: Y. Ito 1729; institutionCode: BKF

#### Potamogeton maackianus

A Benn., 1904

##### Materials

- **Type status:** Other material. **Occurrence:** recordedBy: Y. Ito; **Location:** country: Myanmar; locality: Shan State; Kalow Village, Yae Aye Kan Dam,; verbatimLatitude: 20° 35' 41'' N; verbatimLongitude: 96° 31' 46'' E; **Event:** eventDate: Nov. 26, 2008; **Record Level:** collectionID: Nb. Tanaka et al. 080052; institutionCode: TI

##### Distribution

China (North-eastern, Central, Southern [Yunnan]), Japan, Indonesia (Sumatra), Myanmar, Philippines.

#### Potamogeton lucens

L., 1753

##### Materials

- **Type status:** Other material. **Occurrence:** recordedBy: Y. Ito; **Location:** country: Myanmar; locality: Shan State; naung Shwe, Inlay Lake; verbatimLatitude: 20° 36' N; verbatimLongitude: 96° 54' 44" E; **Event:** eventDate: May. 2, 2005; **Record Level:** collectionID: K. Khaing 05153; institutionCode: TI
- **Type status:** Other material. **Occurrence:** recordedBy: Y. Ito; **Location:** country: Myanmar; locality: Shan State, Pindaya, Inlay Lake; verbatimLatitude: 20° 59' 57'' N; verbatimLongitude: 96° 39' 59'' E; **Event:** eventDate: Dec. 1, 2008; **Record Level:** collectionID: Nb. Tanaka et al. 080630; institutionCode: TI
- **Type status:** Other material. **Occurrence:** recordedBy: Y. Ito; **Location:** country: Myanmar; locality: Inle Lake, Shan State; verbatimLatitude: 16° 53' 19" N; verbatimLongitude: 95° 52' 29" E; **Event:** eventDate: Dec. 3, 2008; **Record Level:** collectionID: Nb. Tanaka et al. 080640; institutionCode: TI

##### Distribution

China (North-eastern, Central, Southern [Yunnan]), Japan, Myanmar, Nepal, Philippines; Europe.

#### Potamogeton nodosus

Poir., 1816

##### Materials

- **Type status:** Other material. **Occurrence:** recordedBy: Y. Ito; **Location:** country: Myanmar; locality: Sagaing Division; Shweuyaung; verbatimLatitude: 24° 31' 2" N; verbatimLongitude: 95° 23' 36" E; **Event:** eventDate: May. 1, 1938; **Record Level:** collectionID: F. G. Dickason 9374; institutionCode: GH
- **Type status:** Other material. **Occurrence:** recordedBy: Y. Ito; **Location:** country: Myanmar; locality: Mandalay Division; verbatimLatitude: 20° 48' N; verbatimLongitude: 95° 15' E; **Event:** eventDate: Nov. 29, 2000; **Record Level:** collectionID: J. Murata et al. 020914; institutionCode: TI
- **Type status:** Other material. **Occurrence:** recordedBy: Y. Ito; **Location:** country: Myanmar; locality: Chiang Mai Province; Chiang Dao District, Ban Bing kong, Bing Kong subdistrict, in the Bing (Ping) river; verbatimLatitude: 19° 36' 9" N; verbatimLongitude: 98° 55' 49" E; **Event:** eventDate: Dec. 6, 1992; **Record Level:** collectionID: J.F. Maxwell 92-803; institutionCode: AAU
- **Type status:** Other material. **Occurrence:** recordedBy: Y. Ito; **Location:** country: Thailand; locality: Chiang Mai; old Mae Ping (Bing) dam, along the Ping (Bing) river; verbatimLatitude: 19° 36' 9" N; verbatimLongitude: 98° 55' 49" E; **Event:** eventDate: Jul. 6, 1995; **Record Level:** collectionID: P. Thavoeburum 1; institutionCode: GH
- **Type status:** Other material. **Occurrence:** recordedBy: Y. Ito; **Location:** country: Thailand; locality: Chiang Mai; Ban Bing kong, Bing Kong subdistrict, in the Bing (Ping) river; verbatimLatitude: 19° 36' 9" N; verbatimLongitude: 98° 55' 49" E; **Event:** eventDate: Dec. 6, 1992; **Record Level:** collectionID: J.F. Maxwell 92-605; institutionCode: GH
- **Type status:** Other material. **Occurrence:** recordedBy: Y. Ito; **Location:** country: Thailand; locality: Chiang Mai Province; Mae Dtang District, in the Bing (Ping) river; verbatimLatitude: 19° 36' 9" N; verbatimLongitude: 98° 55' 49" E; **Event:** eventDate: Dec. 11, 1992; **Record Level:** collectionID: J.F. Maxwell 92-832; institutionCode: AAU
- **Type status:** Other material. **Occurrence:** recordedBy: Y. Ito; **Location:** country: Thailand; locality: Chiang Rai Province; Santonthong, Muang, Lampun; verbatimLatitude: 19° 37' 41" N; verbatimLongitude: 100° 4' 16" E; **Event:** eventDate: Apr. 20, 1971; **Record Level:** collectionID: P. Karnchanomai s.n.; institutionCode: AAU

##### Distribution

Bangladesh, China (North-Eastern, Central [in part], Southern [Yunnan]), India (nationwide), Indonesia (New Guinea, Sumatra, Sulawesi), Myanmar, Nepal, Pakistan, Philippines, Sri Lanka, Thailand, Vietnam; Africa, Europe, America.

#### Potamogeton octandrus

Poir., 1816

##### Materials

- **Type status:** Other material. **Occurrence:** recordedBy: Y. Ito; **Location:** country: Myanmar; locality: Chiang Mai Province; Haung Dong, route to Khan Waang; verbatimLatitude: 18° 42' 58" N; verbatimLongitude: 98° 55' 35" E; **Event:** eventDate: Apr. 5, 1978; **Record Level:** collectionID: T. Smitinand s.n.; institutionCode: BKF
- **Type status:** Other material. **Occurrence:** recordedBy: Y. Ito; **Location:** country: Thailand; locality: Kachin State; between Khalone Village and Shinbweyan; verbatimLatitude: 26° 40' 29'' N; verbatimLongitude: 96° 16' 33'' E; **Event:** eventDate: Dec. 5, 2005; **Record Level:** collectionID: J. Murata et al. 040649; institutionCode: TI

##### Distribution

Bangladesh, Bhutan, China (nationwide), Japan, India (Northern, Eastern, Southern [in part]), Indonesia (Java), Myanmar, Nepal, Papua New Guinea, Thailand, Vietnam; Africa; Oceania.

##### Notes

Myanmar (Ito et al. 2009); Thailand (Ito 2013b).

#### Potamogeton wrightii

Morong, 1886

##### Materials

- **Type status:** Other material. **Occurrence:** recordedBy: Y. Ito; **Location:** country: Myanmar; locality: Mandalay Division; Kyet Mank Taung Dam, Kyaukpadaung Township, Popa; verbatimLatitude: 20° 48' N; verbatimLongitude: 95° 15' E; **Event:** eventDate: Mar. 4, 2003; **Record Level:** collectionID: Kuroiwa et al. 028702; institutionCode: TI
- **Type status:** Other material. **Occurrence:** recordedBy: Y. Ito; **Location:** country: Thailand; locality: Songkla Province; Satingpra District, Bantom island, Lake Songkla, ca. 100 m offshore.; verbatimLatitude: 7° 28' N; verbatimLongitude: 100° 24' E; **Event:** eventDate: Sep. 22, 1984; **Record Level:** collectionID: J.F. Maxwell 84-233; institutionCode: AAU

##### Distribution

Bangradesh, China (nationwide), India (Northern), Indonesia (Celebes, Moluccas, Lonbok, Sumatra), Japan, Malaysia (Borneo, Peninsular), Myanmar, Pakistan, Philippines, Thailand, Vietnam.

#### Stuckenia

Börner, 1912

#### Stuckenia pectinata

(L.) Börner, 1912

##### Materials

- **Type status:** Other material. **Occurrence:** recordedBy: Y. Ito; **Location:** country: Myanmar; locality: Shan State; Inlay Lake; verbatimLatitude: 20° 36' N; verbatimLongitude: 96° 54' 44" E; **Event:** eventDate: Nov. 13, 2005; **Record Level:** collectionID: K. Khaing 05210; institutionCode: TI

##### Distribution

Bangladesh, Bhutan, China (nationwide), India (nationwide), Indonesia (Sumatra, Sulawesi), Japan, Myanmar, Nepal, Pakistan, Sri Lanka; Worldwide.

#### Ruppiaceae

#### Ruppia

L., 1753

#### Ruppia maritima

L., 1753

##### Materials

- **Type status:** Other material. **Occurrence:** recordedBy: Y. Ito; **Location:** country: Thailand; locality: Khao San Yot Natl Park.; verbatimLatitude: 12° 9' 15" N; verbatimLongitude: 99° 58' 44" E; **Event:** eventDate: Nov. 13, 2012; **Record Level:** collectionID: Y. Ito 1706; institutionCode: BKF

##### Distribution

Bangladesh, China (North-Eastern, Central, Southern), India (Southern), ?Myanmar, Nepal, Sri Lanka, Thailand, Vietnam; Worldwide.

#### Asparagales

#### Amaryllidaceae

#### Crinum

L., 1753

#### Crinum thaianum

J. Schul., 1971

##### Materials

- **Type status:** Other material. **Occurrence:** recordedBy: Y. Ito; **Location:** country: Thailand; locality: Phang Nga Province; Klong Tam Nung; verbatimLatitude: 8° 27' 22" N; verbatimLongitude: 98° 31' 8" E; **Record Level:** collectionID: C. Niyomdham 1256; institutionCode: AAU
- **Type status:** Other material. **Occurrence:** recordedBy: Y. Ito; **Location:** country: Thailand; locality: Phang Nga Province; Ban Nang Yon, 30 miles N of Takua Pa, on the way to Ranong; verbatimLatitude: 9° 12' 20" N; verbatimLongitude: 98° 25' 28" E; **Event:** eventDate: Apr. 18, 1970; **Record Level:** collectionID: J. Schulze 357; institutionCode: MO
- **Type status:** Holotype. **Occurrence:** recordedBy: Y. Ito; **Location:** country: Thailand; locality: Phang Nga Province; Ban Nang Yon, 30 miles N of Takua Pa, on the way to Ranong; **Event:** eventDate: Apr. 18, 1970; **Record Level:** collectionID: J. Schulze 1011; institutionCode: US

##### Distribution

Thailand.

##### Notes

Lectotype was designated by Lekhak and Yadav (2012).

Fig. 6.

#### Commelinales

#### Pontederiaceae

#### Eichhornia

Kunth, 1843

#### Eichhornia crassipes

(Mart.) Solms, 1883

##### Materials

- **Type status:** Other material. **Occurrence:** recordedBy: Y. Ito; **Location:** country: Myanmar; locality: Rangoon; verbatimLatitude: 16° 49' 37" N; verbatimLongitude: 96° 8' 58" E; **Event:** eventDate: Jan. 2, 1938; **Record Level:** collectionID: F.G. Dickason 6966; institutionCode: GH
- **Type status:** Other material. **Occurrence:** recordedBy: Y. Ito; **Location:** country: Myanmar; locality: Wakema; verbatimLatitude: 16° 36' 18" N; verbatimLongitude: 95° 10' 49" E; **Event:** eventDate: Jan. 7, 1938; **Record Level:** collectionID: F.G. Dickason 7820; institutionCode: GH
- **Type status:** Other material. **Occurrence:** recordedBy: Y. Ito; **Location:** country: Myanmar; locality: Shan State; Inle lake; verbatimLatitude: 20° 32' 2'' N; verbatimLongitude: 96° 53' 53'' E; **Event:** eventDate: Dec. 3, 2008; **Record Level:** collectionID: Tanaka et al. 080645; institutionCode: MBK
- **Type status:** Other material. **Occurrence:** recordedBy: Y. Ito; **Location:** country: Thailand; locality: Songkla Province, Talae Noi Waterfall Reserve, N end of Lake Songkla; verbatimLatitude: 7° 15' N; verbatimLongitude: 100° 26' 16" E; **Event:** eventDate: Dec. 28, 1978; **Record Level:** collectionID: G. Congdon 162; institutionCode: AAU
- **Type status:** Other material. **Occurrence:** recordedBy: Y. Ito; **Location:** country: Thailand; locality: Songkla Province; Talae Noi waterfowl reserve, N end of lake Songkla, near Phattalung; verbatimLatitude: 7° 15' N; verbatimLongitude: 100° 26' 16" E; **Event:** eventDate: Dec. 28, 1978; **Record Level:** collectionID: G. Congdon & C. Hamilton 162; institutionCode: GH

##### Distribution

Native to South America.

#### Monochoria

C. Presl, 1827

#### Monochoria elata

Ridl., 1918

##### Materials

- **Type status:** Other material. **Occurrence:** recordedBy: Y. Ito; **Location:** country: Thailand; locality: Nakhon Si Thamarat Province; Phru Kuan Kleng; verbatimLatitude: 8° 26' 7" N; verbatimLongitude: 99° 57' 45" E; **Event:** eventDate: Jan. 26, 1999; **Record Level:** collectionID: C. Niyomdham 5662; institutionCode: BKF
- **Type status:** Other material. **Occurrence:** recordedBy: Y. Ito; **Location:** country: Thailand; locality: Phatthalung Province; Khuan Khanun; verbatimLatitude: 7° 44' 6" N; verbatimLongitude: 100° 0' 36" E; **Event:** eventDate: Dec. 24, 2006; **Record Level:** collectionID: R. Pooma et al. 6609; institutionCode: BKF
- **Type status:** Other material. **Occurrence:** recordedBy: Y. Ito; **Location:** country: Thailand; locality: Patalung Province; Thale Luang; verbatimLatitude: 7° 36' N; verbatimLongitude: 100° 10' E; **Event:** eventDate: Mar. 21, 1960; **Record Level:** collectionID: L.B. & E.C. Abbe et al. 9698; institutionCode: BKF
- **Type status:** Other material. **Occurrence:** recordedBy: Y. Ito; **Location:** country: Thailand; locality: Saraburi Province.; verbatimLatitude: 9° 16' N; verbatimLongitude: 99° 32' E; **Event:** eventDate: Oct. 11, 1964; **Record Level:** collectionID: T. Smitinand 29879; institutionCode: BKF
- **Type status:** Other material. **Occurrence:** recordedBy: Y. Ito; **Location:** country: Thailand; locality: Chachoengsao Province; near Phanom Sarakhan, 50km E of Chachoengsao; verbatimLatitude: 13° 40' N; verbatimLongitude: 101° 30' E; **Event:** eventDate: Oct. 1, 1984; **Record Level:** collectionID: G. Murata et al. T-37035; institutionCode: BKF

##### Distribution

Malaysia (Peninsular), Thailand.

#### Monochoria hastata

(L.) Solms, 1883

##### Materials

- **Type status:** Other material. **Occurrence:** recordedBy: Y. Ito; **Location:** country: Myanmar; locality: Rangoon; verbatimLatitude: 16° 49' 37" N; verbatimLongitude: 96° 8' 58" E; **Event:** eventDate: Nov. 1, 1937; **Record Level:** collectionID: F.G. Dickason 6665; institutionCode: GH
- **Type status:** Other material. **Occurrence:** recordedBy: Y. Ito; **Location:** country: Myanmar; locality: Rangoon; verbatimLatitude: 16° 49' 37"; verbatimLongitude: 96° 8' 58" E; **Event:** eventDate: Jan. 2, 1938; **Record Level:** collectionID: F.G. Dickason 7135; institutionCode: GH
- **Type status:** Other material. **Occurrence:** recordedBy: Y. Ito; **Location:** country: Myanmar; locality: Shan State; Inle Lake; verbatimLatitude: 20° 35' 21" N; verbatimLongitude: 96° 54' 35" E; **Event:** eventDate: Jan. 5, 1938; **Record Level:** collectionID: F.G. Dickason 7873; institutionCode: GH
- **Type status:** Other material. **Occurrence:** recordedBy: Y. Ito; **Location:** country: Thailand; locality: Sakon Nakhon Province; Nong Han Pond; verbatimLatitude: 17° 15' 16'' N; verbatimLongitude: 104° 9' 43'' E; **Event:** eventDate: Aug. 25, 2001; **Record Level:** collectionID: R. Pooma, W.J.J.O. de Wilde, B.E.E. Duyfjes, V. Chamchumroon, K. Phattarahirankanok 2579; institutionCode: GH
- **Type status:** Other material. **Occurrence:** recordedBy: Y. Ito; **Location:** country: Thailand; locality: Chiang Mai Province; Ching Hao District, Doi Ching Dao; verbatimLatitude: 19° 22' 7" N; verbatimLongitude: 98° 56' 47" E; **Event:** eventDate: Sep. 30, 1989; **Record Level:** collectionID: J.F. Maxwell 89-1153; institutionCode: GH
- **Type status:** Other material. **Occurrence:** recordedBy: Y. Ito; **Location:** country: Thailand; locality: Lampang Province; Chae Hom District, N part of the forest at Jaehomwittaya school; verbatimLatitude: 18° 41' N; verbatimLongitude: 99° 38' E; **Event:** eventDate: Aug. 6, 2000; **Record Level:** collectionID: M. Panatkool 316; institutionCode: GH
- **Type status:** Other material. **Occurrence:** recordedBy: Y. Ito; **Location:** country: Thailand; locality: Rayong Province; verbatimLatitude: 12° 41' 38" N; verbatimLongitude: 101° 13' 49" E; **Event:** eventDate: Aug. 21, 1977; **Record Level:** collectionID: C. Phengklai et al. 3782; institutionCode: GH
- **Type status:** Other material. **Occurrence:** recordedBy: Y. Ito; **Location:** country: Thailand; locality: Chiang Mai Province; Phek Khaeng Kai; verbatimLatitude: 18° 47' 30" N; verbatimLongitude: 98° 57' 38" E; **Event:** eventDate: Feb. 20, 1991; **Record Level:** collectionID: R. Pooma 91033; institutionCode: GH
- **Type status:** Other material. **Occurrence:** recordedBy: Y. Ito; **Location:** country: Thailand; locality: Narathiwat Province; Paa Wai, Su Ngai Paadee; verbatimLatitude: 6° 7' 37" N; verbatimLongitude: 101° 54' 47" E; **Event:** eventDate: Aug. 24, 1988; **Record Level:** collectionID: C. Niyomdham & W. Ueachirakan 1910; institutionCode: AAU
- **Type status:** Other material. **Occurrence:** recordedBy: Y. Ito; **Location:** country: Thailand; locality: Kalasin Province; Muang Kalasin District.; verbatimLatitude: 16° 20' N; verbatimLongitude: 103° 32' E; **Event:** eventDate: Mar. 13, 1990; **Record Level:** collectionID: C. Phengklai 0077; institutionCode: BKF
- **Type status:** Other material. **Occurrence:** recordedBy: Y. Ito; **Location:** country: Thailand; locality: Phatthalung Province;nok Nam Thale Noi Bird Sanctuary; verbatimLatitude: 7° 47' N; verbatimLongitude: 100° 7' E; **Event:** eventDate: Sep. 1, 1996; **Record Level:** collectionID: P. Wilkin 845; institutionCode: BKF
- **Type status:** Other material. **Occurrence:** recordedBy: Y. Ito; **Location:** country: Thailand; locality: Sakon Nakhon Province; Nong han pond.; verbatimLatitude: 17° 15' 16" N; verbatimLongitude: 104° 9' 43" E; **Event:** eventDate: Aug. 25, 2001; **Record Level:** collectionID: R. Pooma et al. 2579; institutionCode: BKF
- **Type status:** Other material. **Occurrence:** recordedBy: Y. Ito; **Location:** country: Thailand; locality: Rayong Province.; verbatimLatitude: 12° 41' 38" N; verbatimLongitude: 101° 13' 49" E; **Event:** eventDate: Aug. 21, 1977; **Record Level:** collectionID: C. Phengklai et al. 71314; institutionCode: BKF
- **Type status:** Other material. **Occurrence:** recordedBy: Y. Ito; **Location:** country: Thailand; locality: Krungtep Phra Nakhon Province; Soi Somprasong.; verbatimLatitude: 13° 46' 36" N; verbatimLongitude: 100° 30' E; **Event:** eventDate: Jan. 31, 1966; **Record Level:** collectionID: N. Fukuoka 7075; institutionCode: BKF

##### Distribution

Bhutan, Bangladesh, ?Cambodia, China (South), India (nationwide), Myanmar, Nepal, Papua New Guinea, Sri Lanka, Thailand, ?Vietnam.

##### Notes

Fig. 7.

#### Monochoria vaginalis

(Burm.f.) C. Presl ex Kunth, 1843

##### Materials

- **Type status:** Other material. **Occurrence:** recordedBy: Y. Ito; **Location:** country: Myanmar; locality: Shan State; Inlay Lake, Pindaya; verbatimLatitude: 20° 59' 57" N; verbatimLongitude: 96° 39' 59" E; **Event:** eventDate: Dec. 1, 2008; **Record Level:** collectionID: Tanaka et al. 080637; institutionCode: MBK
- **Type status:** Other material. **Occurrence:** recordedBy: Y. Ito; **Location:** country: Myanmar; locality: Rangoon; verbatimLatitude: 16° 49' 37" N; verbatimLongitude: 96° 8' 58" E; **Event:** eventDate: Jan. 10, 1937; **Record Level:** collectionID: F.G. Dickason 6538; institutionCode: GH
- **Type status:** Other material. **Occurrence:** recordedBy: Y. Ito; **Location:** country: Myanmar; locality: Kachin State; Tanaing, Hukaung valley; verbatimLatitude: 26° 6' 34'' N; verbatimLongitude: 96° 42' 58'' E; **Event:** eventDate: Sep. 19, 2005; **Record Level:** collectionID: Tanaka et al. 040481; institutionCode: TI
- **Type status:** Other material. **Occurrence:** recordedBy: Y. Ito; **Location:** country: Myanmar; locality: Shan State; Yae Aye Kan; verbatimLatitude: 20° 35' 41'' N; verbatimLongitude: 96° 31' 46'' E; **Event:** eventDate: Nov. 26, 2008; **Record Level:** collectionID: Tanaka et al. 080056; institutionCode: TI
- **Type status:** Other material. **Occurrence:** recordedBy: Y. Ito; **Location:** country: Thailand; locality: Narathiwat Province; Paa Waai, Su Ngi Paadee; verbatimLatitude: 6° 7' 37" N; verbatimLongitude: 101° 54' 52" E; **Event:** eventDate: Sep. 4, 1987; **Record Level:** collectionID: C. Niyomdham & D. Sriboonma 1543; institutionCode: AAU
- **Type status:** Other material. **Occurrence:** recordedBy: Y. Ito; **Location:** country: Thailand; locality: Narathiwat Province; Kok Kun Bet, Tak Bai; verbatimLatitude: 6° 15' 31" N; verbatimLongitude: 102° 0' 6" E; **Event:** eventDate: Feb. 17, 1988; **Record Level:** collectionID: C. Niyomdham 1698; institutionCode: AAU
- **Type status:** Other material. **Occurrence:** recordedBy: Y. Ito; **Location:** country: Thailand; locality: Trat Province; Saphan Hin; verbatimLatitude: 12° 0' N; verbatimLongitude: 102° 40' E; **Event:** eventDate: May. 3, 1974; **Record Level:** collectionID: R. Geesink et al. 6510; institutionCode: AAU
- **Type status:** Other material. **Occurrence:** recordedBy: Y. Ito; **Location:** country: Thailand; locality: Chanthabury Province; verbatimLatitude: 13° 3' N; verbatimLongitude: 101° 56' E; **Event:** eventDate: Oct. 17, 1971; **Record Level:** collectionID: J.F. Maxwell 71-553; institutionCode: AAU
- **Type status:** Other material. **Occurrence:** recordedBy: Y. Ito; **Location:** country: Thailand; locality: Chonbury Province, Sattahip District; taang Brang; verbatimLatitude: 12° 40' N; verbatimLongitude: 100° 55' E; **Event:** eventDate: Dec. 4, 1972; **Record Level:** collectionID: J.F. Maxwell 72-600; institutionCode: AAU
- **Type status:** Other material. **Occurrence:** recordedBy: Y. Ito; **Location:** country: Thailand; locality: Chonburi Province; Siricha District, Kow Kieo; verbatimLatitude: 13° 14' 15" N; verbatimLongitude: 101° 2' 32" E; **Event:** eventDate: Sep. 4, 1975; **Record Level:** collectionID: J.F. Maxwell 75-979; institutionCode: AAU
- **Type status:** Other material. **Occurrence:** recordedBy: Y. Ito; **Location:** country: Thailand; locality: Songkla Province; Haad Yai District, Klong Hoy Kong, W of Toong Loong; verbatimLatitude: 7° 0' 13" N; verbatimLongitude: 100° 27' 25" e; **Event:** eventDate: May. 30, 1985; **Record Level:** collectionID: J.F. Maxwell 85-553; institutionCode: AAU
- **Type status:** Other material. **Occurrence:** recordedBy: Y. Ito; **Location:** country: Thailand; locality: Songkhla Province; verbatimLatitude: 6° 37' N; verbatimLongitude: 100° 56' E; **Event:** eventDate: Oct. 8, 1988; **Record Level:** collectionID: K. Larsen & S.S. Larsen 10290; institutionCode: AAU
- **Type status:** Other material. **Occurrence:** recordedBy: Y. Ito; **Location:** country: Thailand; locality: Uttaradit Province; Road 1047 from Nam Pat ro Boh Bia; verbatimLatitude: 17° 43' N; verbatimLongitude: 100° 36' E; **Event:** eventDate: Sep. 21, 1996; **Record Level:** collectionID: P.C. Boyce 1121; institutionCode: AAU
- **Type status:** Other material. **Occurrence:** recordedBy: Y. Ito; **Location:** country: Thailand; locality: Nong Khai Province; Se Ka District, Tha Sa-sd; verbatimLatitude: 18° 19' 59'' N; verbatimLongitude: 103° 43' 39'' E; **Event:** eventDate: Aug. 28, 2001; **Record Level:** collectionID: R. Pooma, W.J.J.O. de Wilde, B.E.E. Duyfjes, V. Chamchumroon, K. Phattarahirankanok 2874; institutionCode: GH
- **Type status:** Other material. **Occurrence:** recordedBy: Y. Ito; **Location:** country: Thailand; locality: Phang Nga Province; Koh Kaw Khao island; verbatimLatitude: 9° 0' N; verbatimLongitude: 98° 50' E; **Event:** eventDate: Jul. 15, 1972; **Record Level:** collectionID: K. Larsen et al. 30991; institutionCode: AAU
- **Type status:** Other material. **Occurrence:** recordedBy: Y. Ito; **Location:** country: Thailand; locality: Chaiyaphum Province; Dat Don; verbatimLatitude: 15° 57' N; verbatimLongitude: 102° 2' E; **Event:** eventDate: Aug. 13, 1972; **Record Level:** collectionID: K. Larsen et al. 31774; institutionCode: AAU
- **Type status:** Other material. **Occurrence:** recordedBy: Y. Ito; **Location:** country: Thailand; locality: Ranong Province; NW of Phato; verbatimLatitude: 9° 50' N; verbatimLongitude: 98° 40' E; **Event:** eventDate: May. 2, 1974; **Record Level:** collectionID: K. Larsen & S.S. Larsen 33543; institutionCode: AAU
- **Type status:** Other material. **Occurrence:** recordedBy: Y. Ito; **Location:** country: Thailand; locality: Maehongson Province; Khun Yuam; verbatimLatitude: 18° 15' N; verbatimLongitude: 98° 0' E; **Event:** eventDate: Sep. 7, 1974; **Record Level:** collectionID: K. Larsen & S.S. Larsen 34249; institutionCode: AAU
- **Type status:** Other material. **Occurrence:** recordedBy: Y. Ito; **Location:** country: Thailand; locality: Phitsanulok Province; Tung Salaeng Luang; verbatimLatitude: 16° 59' N; verbatimLongitude: 100° 53' E; **Event:** eventDate: Jul. 24, 1966; **Record Level:** collectionID: K. Larsen et al. 833; institutionCode: AAU
- **Type status:** Other material. **Occurrence:** recordedBy: Y. Ito; **Location:** country: Thailand; locality: Phuket Province; W of Ban Bo Han; verbatimLatitude: 8° 7' N; verbatimLongitude: 98° 18' E; **Event:** eventDate: Oct. 8, 1970; **Record Level:** collectionID: C. Charoenphol et al. 3422; institutionCode: AAU
- **Type status:** Other material. **Occurrence:** recordedBy: Y. Ito; **Location:** country: Thailand; locality: Mahasarakarm Province; Koksung District, Amphur Chiengyeun; verbatimLatitude: 16° 11' 18" N; verbatimLongitude: 103° 17' 58" E; **Event:** eventDate: Sep. 18, 1984; **Record Level:** collectionID: N. Fukuoka T-36166; institutionCode: GH
- **Type status:** Other material. **Occurrence:** recordedBy: Y. Ito; **Location:** country: Thailand; locality: Chiang Mai Province; Doi Inthanon, N side of the route RS-13; verbatimLatitude: 18° 32' 23" N; verbatimLongitude: 98° 31' 15" E; **Event:** eventDate: Jul. 22, 1988; **Record Level:** collectionID: S. Tsugaru T-61719; institutionCode: GH
- **Type status:** Other material. **Occurrence:** recordedBy: Y. Ito; **Location:** country: Thailand; locality: Chachoengsao Province; Chachoengsao Wildlife Reserve; verbatimLatitude: 13° 26' N; verbatimLongitude: 101° 55' E; **Event:** eventDate: Sep. 11, 1999; **Record Level:** collectionID: D.J. middleton, W. Sangkamethawee 227; institutionCode: GH
- **Type status:** Other material. **Occurrence:** recordedBy: Y. Ito; **Location:** country: Thailand; locality: Krabi Province; Klongtom District, Ban Klong Rat; verbatimLatitude: 7° 55' 48" N; verbatimLongitude: 99° 13' 42" E; **Event:** eventDate: Nov. 29, 1986; **Record Level:** collectionID: J.F. Maxwell 86-991; institutionCode: GH
- **Type status:** Other material. **Occurrence:** recordedBy: Y. Ito; **Location:** country: Thailand; locality: Petchabury; verbatimLatitude: 13° 30' 6" N; verbatimLongitude: 99° 47' 37" E; **Event:** eventDate: 11-14-12; **Record Level:** institutionCode: BKF
- **Type status:** Other material. **Occurrence:** recordedBy: Y. Ito; **Location:** country: Vietnum; locality: Kontum Province; verbatimLatitude: 14° 42' 40" N; verbatimLongitude: 107° 49' 48" E; **Event:** eventDate: Apr. 14, 1995; **Record Level:** collectionID: L. Averyanov et al.VH1360; institutionCode: AAU
- **Type status:** Other material. **Occurrence:** recordedBy: Y. Ito; **Location:** country: Vietnum; locality: Kon Tum Province; Dak Gley District; verbatimLatitude: 15° 6' 57" N; verbatimLongitude: 107° 42' 55" E; **Event:** eventDate: Nov. 22, 1995; **Record Level:** collectionID: L. Averyanov et al. VH1906; institutionCode: AAU
- **Type status:** Other material. **Occurrence:** recordedBy: Y. Ito; **Location:** country: Vietnum; locality: Lam Dong Province; Lac Dung District; verbatimLatitude: 12° 8' N; verbatimLongitude: 108° 39' E; **Event:** eventDate: Apr. 9, 1997; **Record Level:** collectionID: L. Averyanov et al. VH3814; institutionCode: AAU
- **Type status:** Other material. **Occurrence:** recordedBy: Y. Ito; **Location:** country: Vietnum; locality: Lam Dong Province; Lac Duong District; verbatimLatitude: 12° 8' N; verbatimLongitude: 108° 39' E; **Event:** eventDate: Apr. 21, 1997; **Record Level:** collectionID: L. Averyanov et al. VH3896; institutionCode: AAU

##### Distribution

Bangladesh, Bhutan, China (nationwide), India (nationwide), ?Indonesia, Japan, Korea, ?Malaysia, Myanmar, Nepal, Papua New Guinea, Pakistan, Philippines, Sri Lanka, Thailand; Africa; Oceania.

#### Typhaceae

#### Typha

L., 1753

#### Typha angustifolia

L., 1753

##### Materials

- **Type status:** Other material. **Occurrence:** recordedBy: Y. Ito; **Location:** country: Thailand; locality: Chantabury Province; Klang (between bangkok and Paknam); verbatimLatitude: 12° 16' N; verbatimLongitude: 102° 20' E; **Event:** eventDate: Mar. 11, 1958; **Record Level:** collectionID: Kai Larsen 2053; institutionCode: GH
- **Type status:** Other material. **Occurrence:** recordedBy: Y. Ito; **Location:** country: Thailand; locality: Chonburi Province; Siracha District, Siracha island; verbatimLatitude: 13° 8' 56" N; verbatimLongitude: 100° 48' 37" E; **Event:** eventDate: Nov. 29, 1992; **Record Level:** collectionID: J.F. Maxwell 92-776; institutionCode: GH
- **Type status:** Other material. **Occurrence:** recordedBy: Y. Ito; **Location:** country: Thailand; locality: Petchabury; verbatimLatitude: 13° 24' 30" N; verbatimLongitude: 99° 48' 44" E; **Event:** eventDate: Nov. 14, 2012; **Record Level:** collectionID: Y. Ito 1708; institutionCode: BKF
- **Type status:** Other material. **Occurrence:** recordedBy: Y. Ito; **Location:** country: Thailand; verbatimLatitude: 13° 45' N; verbatimLongitude: 100° 29' E; **Record Level:** collectionID: M. Tagawa & I. Yamada T 112; institutionCode: TI

##### Distribution

Bangladesh, China (nationwide), India (nationwide), Japan, ?Myanmar, Pakistan; Worldwide.

#### Poales

#### Eriocaulaceae

#### Eriocaulon

L., 1753

#### Eriocaulon setaceum

L., 1753

##### Materials

- **Type status:** Other material. **Occurrence:** recordedBy: Y. Ito; **Location:** country: Thailand; locality: Sakon Nakhon; Thamphura Fall; verbatimLatitude: 17° 9' 15'' N; verbatimLongitude: 104° 8' 10'' E; **Event:** eventDate: Jul. 22, 2003; **Record Level:** collectionID: Th. Wongprasert et al. 037-15; institutionCode: BKF
- **Type status:** Other material. **Occurrence:** recordedBy: Y. Ito; **Location:** country: Thailand; locality: Phangnga, Khuraburi, Ko Phrathong; verbatimLatitude: 9° 6' N; verbatimLongitude: 98° 17' E; **Event:** eventDate: Aug. 24, 2005; **Record Level:** collectionID: C. Phengklai et al. 15067; institutionCode: BKF
- **Type status:** Other material. **Occurrence:** recordedBy: Y. Ito; **Location:** country: Thailand; locality: Phu Kradung Natl Park. Tambon Srithan, Phu Kradung District; decimalLatitude: 16.90 N; decimalLongitude: 101.80 E; **Event:** eventDate: Nov. 1, 2002; **Record Level:** collectionID: A. prajaksood and S. Suddee 243; institutionCode: BKF
- **Type status:** Other material. **Occurrence:** recordedBy: Y. Ito; **Location:** country: Thailand; locality: Tambon Putthawee, Makham district.; **Event:** eventDate: Oct. 5, 2002; **Record Level:** collectionID: A. prajaksood and S. Suddee 171; institutionCode: BKF
- **Type status:** Other material. **Occurrence:** recordedBy: Y. Ito; **Location:** country: Thailand; locality: Khao Yai Natl Park, Muang District.; decimalLatitude: 14° 26' 29" N; decimalLongitude: 101° 22' 11" E; **Event:** eventDate: Sep. 28, 2002; **Record Level:** collectionID: A. prajaksood and S. Suddee 163; institutionCode: BKF
- **Type status:** Other material. **Occurrence:** recordedBy: Y. Ito; **Location:** country: Thailand; locality: Sakon Nakhon; Phu Phan Natl Park.; verbatimLatitude: 16° 51' N; verbatimLongitude: 103° 55' E; **Event:** eventDate: Jul. 4, 1999; **Record Level:** collectionID: A. prajaksood 155; institutionCode: BKF

##### Distribution

Bangladesh, ?Cambodia, China (Southern), India (Western, Southern), Japan, ?Laos, ?Myanmar, Sri Lanka, Thailand, ?Vietnam.

#### Ceratophyllales

#### Ceratophyllaceae

#### Ceratophyllum

L., 1753

#### Ceratophyllum demersum

L., 1753

##### Materials

- **Type status:** Other material. **Occurrence:** recordedBy: Y. Ito; **Location:** country: Myanmar; verbatimLatitude: 16° 53' 19" N; verbatimLongitude: 95° 52' 29" E; **Record Level:** collectionID: TI040058; institutionCode: TI
- **Type status:** Other material. **Occurrence:** recordedBy: Y. Ito; **Location:** country: Myanmar; verbatimLatitude: 16° 53' 19.18"; verbatimLongitude: 95° 52' 28.59"; **Record Level:** collectionID: TI05152; institutionCode: TI
- **Type status:** Other material. **Occurrence:** recordedBy: Y. Ito; **Location:** country: Myanmar; **Record Level:** collectionID: MBK080650; institutionCode: TI
- **Type status:** Other material. **Occurrence:** recordedBy: Y. Ito; **Location:** country: Thailand; locality: Kantchanabury; Hotel river Kwai; verbatimLatitude: 14° 1' 59" N; verbatimLongitude: 99° 31' 10" E; **Event:** eventDate: Nov. 15, 2012; **Record Level:** collectionID: Y. Ito 1723; institutionCode: BKF
- **Type status:** Other material. **Occurrence:** recordedBy: Y. Ito; **Location:** country: Thailand; locality: Bangkok; verbatimLatitude: 13° 45' N; verbatimLongitude: 100° 30' E; **Event:** eventDate: Aug. 23, 1926; **Record Level:** collectionID: A. F. G. Kerr 11027; institutionCode: GH
- **Type status:** Other material. **Occurrence:** recordedBy: Y. Ito; **Location:** country: Thailand; locality: Bangkok; verbatimLatitude: 13° 45' N; verbatimLongitude: 100° 30' E; **Event:** eventDate: Aug. 23, 1926; **Record Level:** collectionID: A. Marcan 2135; institutionCode: GH

##### Distribution

Worldwide.

#### Proteales

#### Nelumbonaceae

#### Nelumbo

Adans., 1763

#### Nelumbo nucifera

Gaertn., 1788

##### Distribution

Native to tropics in Asia and Oceania.

#### Saxifragales

#### Haloragaceae

#### Myriophyllum

L., 1753

#### Myriophyllum brasiliense

Cambess, 1829

##### Materials

- **Type status:** Other material. **Occurrence:** recordedBy: Y. Ito; **Location:** country: Thailand; locality: Pahayao Province; 6 km from Phayaoa; verbatimLatitude: 19° 9' N; verbatimLongitude: 99° 56' E; **Event:** eventDate: Sep. 24, 1996; **Record Level:** collectionID: J.A.N. Parnell & D.A. Simpson 1744; institutionCode: AAU

##### Distribution

Native in South America, naturalized in Asia, Oceania, and North America.

#### Myriophyllum spicatum

L., 1753

##### Materials

- **Type status:** Other material. **Occurrence:** recordedBy: Y. Ito; **Location:** country: Myanmar; verbatimLatitude: 16° 53' 19" N; verbatimLongitude: 95° 52' 29" E; **Record Level:** institutionCode: TI

##### Distribution

China (nationwide), Japan, Myanmar; Europe.

#### Myriophyllum tetrandrum

Roxb., 1820

##### Materials

- **Type status:** Other material. **Occurrence:** recordedBy: Y. Ito; **Location:** country: Thailand; locality: Rayong Province; Ban phe; verbatimLatitude: 12° 40' N; verbatimLongitude: 101° 25' E; **Event:** eventDate: Dec. 16, 1974; **Record Level:** collectionID: R. Geesink, P. Hiepko 7874; institutionCode: BKF
- **Type status:** Other material. **Occurrence:** recordedBy: Y. Ito; **Location:** country: Thailand; locality: Chumphon, Ma Chana; verbatimLatitude: 10° 0' 24" N; verbatimLongitude: 99° 3' 18" E; **Event:** eventDate: Mar. 4, 1976; **Record Level:** collectionID: T. Gunlund 12127; institutionCode: BKF
- **Type status:** Other material. **Occurrence:** recordedBy: Y. Ito; **Location:** country: Thailand; locality: ChaChoengsao Province; Khao Ang Rua Nai Wildlife Sanctuary; verbatimLatitude: 13° 15' N; verbatimLongitude: 101° 50' E; **Event:** eventDate: Nov. 5, 1993; **Record Level:** collectionID: K. Larsen et al. 44234; institutionCode: AAU
- **Type status:** Other material. **Occurrence:** recordedBy: Y. Ito; **Location:** country: Thailand; locality: Saraburi Province; Muang District, Sahm Lahn forest; verbatimLatitude: 14° 31' 51" N; verbatimLongitude: 100° 54' 34" E; **Event:** eventDate: Nov. 23, 1974; **Record Level:** collectionID: J.F. Maxwell 74-995; institutionCode: AAU
- **Type status:** Other material. **Occurrence:** recordedBy: Y. Ito; **Location:** country: Thailand; locality: Songkla Province; ca 4km SE of Chana, along hwy 43; verbatimLatitude: 6° 52' 34" N; verbatimLongitude: 100° 57' 11" E; **Event:** eventDate: Jul. 11, 1985; **Record Level:** collectionID: J.F. Maxwell 85-702; institutionCode: AAU
- **Type status:** Other material. **Occurrence:** recordedBy: Y. Ito; **Location:** country: Thailand; locality: Yala Province; verbatimLatitude: 6° 34' N; verbatimLongitude: 101° 18' E; **Event:** eventDate: Oct. 22, 1970; **Record Level:** collectionID: C. Charoenphol et al. 4144; institutionCode: AAU

##### Distribution

Bangladesh, China (Southern), India (nationwide), Malaysia (Peninsular), Myanmar, Thailand, Sri Lanka, ?Vietnam.

#### Myriophyllum tuberculatum

Roxb., 1820

##### Materials

- **Type status:** Other material. **Occurrence:** recordedBy: Y. Ito; **Location:** country: Myanmar; locality: Shan State; verbatimLatitude: 20° 59' 57'' N; verbatimLongitude: 96° 39' 59'' E; **Event:** eventDate: Dec. 1, 2008; **Record Level:** collectionID: Tanaka et al. 080626; institutionCode: TI
- **Type status:** Other material. **Occurrence:** recordedBy: Y. Ito; **Location:** country: Thailand; locality: Pattalung Province; Bah Baun District, Ban Naung Tong; verbatimLatitude: 7° 13' 34" N; verbatimLongitude: 100° 8' 39" E; **Event:** eventDate: Jan. 12, 1987; **Record Level:** collectionID: J.F. Maxwell 87-29; institutionCode: BKF

##### Distribution

Bangladesh, China (Southern), India (Eastern, Southern), Indonesia, Malaysia (Penninsular, Borneo), Myanmar, ?Pakistan, Thailand; Australia.

#### Fabales

#### Fabaceae

#### Neptunia

Lour., 1790

#### Neptunia oleracea

Lour., 1790

##### Materials

- **Type status:** Other material. **Occurrence:** recordedBy: Y. Ito; **Location:** country: Thailand; locality: Hotel river Kwai, Kantchanabury; verbatimLatitude: 14° 1' 59" N; verbatimLongitude: 99° 31' 10" E; **Event:** eventDate: Nov. 15, 2012; **Record Level:** collectionID: Y. Ito 1720; institutionCode: BKF

##### Distribution

India, ?Myanmar, Thailand.

##### Notes

Fig. 8.

#### Myrtales

#### Onagraceae

#### Ludwigia

L., 1753

#### Ludwigia adscendens

(L.) H. Hara, 1953

##### Materials

- **Type status:** Other material. **Occurrence:** recordedBy: Y. Ito; **Location:** country: Thailand; locality: Hotel river Kwai, Kantchanabury; verbatimLatitude: 14° 1' 59" N; verbatimLongitude: 99° 31' 10" E; **Event:** eventDate: Nov. 15, 2012; **Record Level:** collectionID: Y. Ito 1720; institutionCode: BKF
- **Type status:** Other material. **Occurrence:** recordedBy: Y. Ito; **Location:** country: Laos; locality: Savannaket Province; Nakai Plateau, Theun Douan lake, near Phong Sa Vahn resettlement village.; verbatimLatitude: 16° 34' 10" N; verbatimLongitude: 104° 44' 54" E; **Event:** eventDate: May. 4, 2007; **Record Level:** collectionID: J. F. Maxwell 07-313; institutionCode: GH
- **Type status:** Other material. **Occurrence:** recordedBy: Y. Ito; **Location:** country: Myanmar; verbatimLatitude: 16° 53' 19" N; verbatimLongitude: 95° 52' 29" E; **Event:** eventDate: Dec. 8, 2006; **Record Level:** institutionCode: TI
- **Type status:** Other material. **Occurrence:** recordedBy: Y. Ito; **Location:** country: Myanmar; verbatimLatitude: 16° 53' 19"; verbatimLongitude: 95° 52' 28" E; **Event:** eventDate: Dec. 1, 2008; **Record Level:** institutionCode: TI
- **Type status:** Other material. **Occurrence:** recordedBy: Y. Ito; **Location:** country: Thailand; locality: Songkla Province, Talae Noi Waterfowl Reserve, N end of Lake Songkla, near Phattalung.; verbatimLatitude: 7° 15' N; verbatimLongitude: 100° 26' 16" E; **Event:** eventDate: Dec. 28, 1978; **Record Level:** collectionID: G. Congdon & C. Hamilton #155; institutionCode: GH
- **Type status:** Other material. **Occurrence:** recordedBy: Y. Ito; **Location:** country: Thailand; locality: Phetchabury.; verbatimLatitude: 13° 24' 30" N; verbatimLongitude: 99° 48' 44" E; **Event:** eventDate: Nov. 14, 2012; **Record Level:** collectionID: Y. Ito 1709; institutionCode: BKF

##### Distribution

Bangladesh, Cambodia, China (nationwide), India (nationwide), Laos, Myanmar, Nepal, Sri Lanka, Thailand, Vietnam.

#### Caryophyllales

#### Polygonaceae

#### Persicaria

Mill., 1754

#### Persicaria attenuata

(R. Br.) Soják, 1974

#### Persicaria attenuata pulchra

(Blume) K.L. Wilson, 1990

##### Materials

- **Type status:** Other material. **Occurrence:** recordedBy: Y. Ito; **Location:** country: Thailand; locality: Bung Bonapet Nonhunting Area; verbatimLatitude: 15° 41' 40" N; verbatimLongitude: 100° 16' 3" E; **Event:** eventDate: Nov. 16, 2012; **Record Level:** collectionID: Y. Ito 1732; institutionCode: BKF

##### Distribution

China (Southern [Taiwan]), India, Indonesia (Java, Sumatra), ?Japan, Malaysia (Borneo), ?Myanmar, Philippines, Thailand; ?Australia.

##### Notes

Fig. 9.

#### Solanales

#### Convolvulaceae

#### Ipomoea

L., 1753

#### Ipomoea aquatica

Forssk., 1775

##### Materials

- **Type status:** Other material. **Occurrence:** recordedBy: Y. Ito; **Location:** country: Myanmar; verbatimLatitude: 16° 53' 19" N; verbatimLongitude: 95° 52' 29" E; **Record Level:** collectionID: ???; institutionCode: TI
- **Type status:** Other material. **Occurrence:** recordedBy: Y. Ito; **Location:** country: Myanmar; locality: Mandalay Division; in fields, rice paddies etc., a serious weed "Kazun"; verbatimLatitude: 21° 14' 17" n; verbatimLongitude: 95° 52' 33" E; **Event:** eventDate: Jan. 10, 1939; **Record Level:** collectionID: F.G. Dickason 9575; institutionCode: GH
- **Type status:** Other material. **Occurrence:** recordedBy: Y. Ito; **Location:** country: Myanmar; locality: Kachin State; Bhamo district. Habitat Kaugyi; verbatimLatitude: 24° 15' 19" N; verbatimLongitude: 97° 14' 4" E; **Event:** eventDate: Sep. 12, 1908; **Record Level:** collectionID: -; institutionCode: GH
- **Type status:** Other material. **Occurrence:** recordedBy: Y. Ito; **Location:** country: Thailand; locality: Songkla Prov., Ampoe Satingpra, Ban Satingpra; verbatimLatitude: 7° 10' 40" N; verbatimLongitude: 100° 36' 51" E; **Event:** eventDate: Dec. 24, 1978; **Record Level:** collectionID: G. Congdon & C. Hamilton 127; institutionCode: GH
- **Type status:** Other material. **Occurrence:** recordedBy: Y. Ito; **Location:** country: Thailand; locality: Tarutao end of beach at Malacca Creek; verbatimLatitude: 6° 33' 9" N; verbatimLongitude: 99° 40' 8" E; **Event:** eventDate: Nov. 29, 1979; **Record Level:** collectionID: G. Congdon 207; institutionCode: GH
- **Type status:** Other material. **Occurrence:** recordedBy: Y. Ito; **Location:** country: Thailand; locality: Uttradit, Fishai, Bao Bak Klong; verbatimLatitude: 17° 37' 33" N; verbatimLongitude: 100° 6' 16" E; **Event:** eventDate: Oct. 20, 1992; **Record Level:** collectionID: J. F. Maxwell 92-655; institutionCode: GH
- **Type status:** Other material. **Occurrence:** recordedBy: Y. Ito; **Location:** country: Thailand; locality: Nakhon ratchasima Prov., Pak Chong Dist., Khao Yai Park; verbatimLatitude: 14° 39' 31" N; verbatimLongitude: 101° 26' E; **Event:** eventDate: Oct. 17, 1985; **Record Level:** collectionID: G. Staples, T. Wongprassert 162; institutionCode: GH
- **Type status:** Other material. **Occurrence:** recordedBy: Y. Ito; **Location:** country: Thailand; locality: Trang Prov., Yanta Khao Dist.; verbatimLatitude: 7° 26' 19" N; verbatimLongitude: 99° 44' 44" E; **Event:** eventDate: Oct. 24, 1985; **Record Level:** collectionID: G. Staples, T. Wongprassert 180; institutionCode: GH
- **Type status:** Other material. **Occurrence:** recordedBy: Y. Ito; **Location:** country: Thailand; locality: Phuket Prov., near Nai Yang National Park; verbatimLatitude: 8° 5' 19" N; verbatimLongitude: 98° 17' 52" E; **Event:** eventDate: Oct. 25, 1985; **Record Level:** collectionID: G. Staples, T. Wongprassert 200; institutionCode: GH
- **Type status:** Other material. **Occurrence:** recordedBy: Y. Ito; **Location:** country: Thailand; locality: Saraburi Prov.; verbatimLatitude: 14° 32' 56" N; verbatimLongitude: 100° 54' 13" E; **Event:** eventDate: Apr. 11, 1985; **Record Level:** collectionID: G. Staples, T. Wongprassert 219; institutionCode: GH
- **Type status:** Other material. **Occurrence:** recordedBy: Y. Ito; **Location:** country: Thailand; locality: Kanchanaburi Prov.; verbatimLatitude: 14° 2' 46" N; verbatimLongitude: 99° 31' 7" E; **Event:** eventDate: Nov. 15, 1985; **Record Level:** collectionID: G. Staples, T. Wongprassert 274; institutionCode: GH
- **Type status:** Other material. **Occurrence:** recordedBy: Y. Ito; **Location:** country: Thailand; locality: Kampangpet; verbatimLatitude: 7° 11' 31" N; verbatimLongitude: 100° 35' 35" E; **Event:** eventDate: Nov. 28, 1977; **Record Level:** collectionID: C. Phengklai et al. 3944; institutionCode: GH
- **Type status:** Other material. **Occurrence:** recordedBy: Y. Ito; **Location:** country: Thailand; locality: Khon Kaen Prov., Chumphae Distr.; verbatimLatitude: 16° 33' 9" N; verbatimLongitude: 102° 5' 50" E; **Event:** eventDate: May. 12, 1985; **Record Level:** collectionID: G. Staples, T. Wongprassert 399; institutionCode: GH
- **Type status:** Other material. **Occurrence:** recordedBy: Y. Ito; **Location:** country: Thailand; locality: Songkla, Ranot; verbatimLatitude: 7° 47' 31" N; verbatimLongitude: 100° 18' 13" E; **Event:** eventDate: Dec. 22, 2006; **Record Level:** collectionID: Pooma, R., Pattharahirantricin, N., Sirimongkol, S. 6548; institutionCode: GH

##### Distribution

Bangladesh, Cambodia, China (nationwide), India (nationwide), Laos, Myanmar, Nepal, Sri Lanka, Thailand, Vietnam.

#### Plantaginaceae

#### Callitriche

L., 1753

#### Callitriche palustris

L., 1753

##### Distribution

Bangladesh, Bhutan, China (nationwide), India (Northern [Jammu and Kashmir], Southern [Tamil Nadu]), Japan, ?Myanmar, Nepal, Pakistan, Sri Lanka; Cosmopolitan.

#### Limnophila

R. Br., 1810

#### Limnophila heterophylla

(L.) Druce, 1914

##### Materials

- **Type status:** Other material. **Occurrence:** recordedBy: Y. Ito; **Location:** country: Thailand; locality: Chiang Rai Province; Mae Chan District, Akcha village; verbatimLatitude: 20° 12' 4" N; verbatimLongitude: 99° 56' 45" E; **Event:** eventDate: Nov. 7, 1993; **Record Level:** collectionID: J.F. Maxwell 93-1364; institutionCode: GH
- **Type status:** Other material. **Occurrence:** recordedBy: Y. Ito; **Location:** country: Thailand; locality: Chiang Rai Province; Maesai District, Wat Thamsaohin Phyanak; verbatimLatitude: 20° 24' 30" N; verbatimLongitude: 99° 53' E; **Event:** eventDate: Feb. 14, 1983; **Record Level:** collectionID: H. Koyama, H. Terao, T. Wongprasert T-33500; institutionCode: GH
- **Type status:** Other material. **Occurrence:** recordedBy: Y. Ito; **Location:** country: Thailand; locality: Chiang Mai Province; Doi Inthanon, near guest house; verbatimLatitude: 18° 32' 55" N; verbatimLongitude: 98° 31' 28" E; **Event:** eventDate: Jul. 23, 1988; **Record Level:** collectionID: S. Tsugaru T-61736; institutionCode: GH
- **Type status:** Other material. **Occurrence:** recordedBy: Y. Ito; **Location:** country: Thailand; locality: Saraburi; verbatimLatitude: 14° 34' 51" N; verbatimLongitude: 100° 54' 52" E; **Event:** eventDate: Feb. 28, 1982; **Record Level:** collectionID: A. Ubolcholaket s.n.; institutionCode: AAU
- **Type status:** Other material. **Occurrence:** recordedBy: Y. Ito; **Location:** country: Thailand; locality: Chonburi Province; Sattalip District, Taong Breng; verbatimLatitude: 12° 43' N; verbatimLongitude: 100°56' E; **Event:** eventDate: May. 7, 1972; **Record Level:** collectionID: J.F. Maxwell 72-221; institutionCode: AAU
- **Type status:** Other material. **Occurrence:** recordedBy: Y. Ito; **Location:** country: Thailand; locality: Narathiwat Province; Toh Deang peat swamp; verbatimLatitude: 6° 25' 35" N; verbatimLongitude: 101° 49' 26" E; **Event:** eventDate: Aug. 28, 2001; **Record Level:** collectionID: C. Niyomdham 6533; institutionCode: BKF

##### Distribution

Cambodia, China (Eastern), India, Malaysia, ?Myanmar, Nepal, Sri Lanka, Thailand, Vietnam.

#### Limnophila indica

(Roxb.) Benth., 1835

##### Materials

- **Type status:** Other material. **Occurrence:** recordedBy: Y. Ito; **Location:** country: Myanmar; locality: Shan State; verbatimLatitude: 20° 59' 57'' N; verbatimLongitude: 96° 39' 59'' E; **Event:** eventDate: Dec. 1, 2008; **Record Level:** collectionID: Tanaka et al. 080633; institutionCode: TI
- **Type status:** Other material. **Occurrence:** recordedBy: Y. Ito; **Location:** country: Thailand; locality: Loei Province; Phu Kradung, S. of Loi; verbatimLatitude: 16° 53' N; verbatimLongitude: 101° 53' E; **Event:** eventDate: Nov. 7, 1970; **Record Level:** collectionID: Ch. Charoenphol et al. 4797; institutionCode: AAU
- **Type status:** Other material. **Occurrence:** recordedBy: Y. Ito; **Location:** country: Thailand; locality: Kanchanaburi Province; Thong Pha Phum District, Rintin Forest; verbatimLatitude: 14° 47' 41" N; verbatimLongitude: 98° 44' 42" E; **Event:** eventDate: Nov. 6, 1979; **Record Level:** collectionID: T. Shimizu et al. T-21918; institutionCode: BKF
- **Type status:** Other material. **Occurrence:** recordedBy: Y. Ito; **Location:** country: Thailand; locality: Mukdahan Province; Muang District, Dongman Village, along route 212; verbatimLatitude: 16° 32' 16" N; verbatimLongitude: 104° 42' 16" E; **Event:** eventDate: Dec. 12, 1982; **Record Level:** collectionID: H. Koyama et al. T-30856; institutionCode: BKF
- **Type status:** Other material. **Occurrence:** recordedBy: Y. Ito; **Location:** country: Thailand; locality: Khon Kaen Province; Phu Khieo Game reserve, 80 km E of Phetchabun; verbatimLatitude: 16° 28' 32" N; verbatimLongitude: 102° 50' 4" E; **Event:** eventDate: Nov. 7, 1984; **Record Level:** collectionID: G. Murata T-41716; institutionCode: BKF

##### Distribution

Bangladesh, ?Cambodia, China (South), India (nationwide), ?Indonesia, Japan, ?Laos, ?Malaysia, Myanmar, ?Nepal, Pakistan, Papua New Guinea, Sri Lanka, Thailand, ?Vietnam; Africa; Oceania.

#### Limnophila sessiliflora

(Vahl) Blume, 1826

##### Materials

- **Type status:** Other material. **Occurrence:** recordedBy: Y. Ito; **Location:** country: Myanmar; locality: Kachin State; Tawang Hka (river) near Makaw Village, 6 miles N of Tanaing.; verbatimLatitude: 26° 26' 51'' N; verbatimLongitude: 96° 41' 16'' E; **Event:** eventDate: Dec. 2, 2005; **Record Level:** collectionID: Murata et al. 040837; institutionCode: TI

##### Distribution

Bangladesh, Bhutan, ?Cambodia, China (nationwide), India (nationwide), ?Indonesia (Java), Japan, ?Malaysia, Myanmar, Nepal, Sri Lanka, Thailand, ?Vietnam.

#### Phrymaceae

#### Mimulus

L., 1753

#### Mimulus orbicularis

Wall. ex Benth., 1835

##### Materials

- **Type status:** Other material. **Occurrence:** recordedBy: Y. Ito; **Location:** country: Thailand; locality: Ayutthaya Provinve; Pathun Thani, Bangsit.; verbatimLatitude: 14° 18' 39" N; verbatimLongitude: 100° 17' 38" E; **Event:** eventDate: Nov. 14, 1965; **Record Level:** collectionID: M. Tagawa & K. Iwatsuki T-289; institutionCode: BKF, TI

##### Distribution

Bangladesh, India (Western [Orissa, West Bengal]), Myanmar, Thailand; Australia.

##### Notes

Barkera and Jobson (2013) reported a new locality of this species from northern Australia.

#### Lamiales

#### Lentibulariaceae

#### Utricularia

L., 1753

#### Utricularia aurea

Lour., 1790

##### Materials

- **Type status:** Other material. **Occurrence:** recordedBy: Y. Ito; **Location:** country: Myanmar; locality: Inle Lake; verbatimLatitude: 20° 35' 21" N; verbatimLongitude: 96° 54' 35" E; **Event:** eventDate: Jan. 5, 1938; **Record Level:** collectionID: F.G. Dickason 7857; institutionCode: GH
- **Type status:** Other material. **Occurrence:** recordedBy: Y. Ito; **Location:** country: Myanmar; locality: Bago Division; Pyat Township.; verbatimLatitude: 18° 42' 22'' N; verbatimLongitude: 95° 5' 59'' E; **Event:** eventDate: Dec. 9, 2006; **Record Level:** collectionID: Sugawara et al. 036434; institutionCode: TI
- **Type status:** Other material. **Occurrence:** recordedBy: Y. Ito; **Location:** country: Myanmar; locality: Kachin State; Tanaing Township; verbatimLatitude: 26° 6' 34" N; verbatimLongitude: 96° 42' 58" E; **Event:** eventDate: Sep. 19, 2005; **Record Level:** collectionID: Tanaka et al. 040485; institutionCode: TI
- **Type status:** Other material. **Occurrence:** recordedBy: Y. Ito; **Location:** country: Thailand; locality: Chanthaburi Province; Makham; verbatimLatitude: 13° 0' N; verbatimLongitude: 101° 50' E; **Event:** eventDate: Aug. 5, 1973; **Record Level:** collectionID: R. Geesink & C. Phengkhlai 6333; institutionCode: AAU
- **Type status:** Other material. **Occurrence:** recordedBy: Y. Ito; **Location:** country: Thailand; locality: Ayuthia Province; Sara Buri - Klang Dong; verbatimLatitude: 14° 32' 9" N; verbatimLongitude: 100° 54' 34" E; **Event:** eventDate: Aug. 8, 1979; **Record Level:** collectionID: T. Shimizu, H. Toyokuni, H. Koyama, T. Yahara, C. Niyomdham 9479; institutionCode: GH
- **Type status:** Other material. **Occurrence:** recordedBy: Y. Ito; **Location:** country: Thailand; locality: Narathiwat Province; Swamp forest station, N of Sungai Kolok; verbatimLatitude: 6° 4' N; verbatimLongitude: 101° 57' E; **Event:** eventDate: Aug. 17, 1995; **Record Level:** collectionID: K. Larsen et al. 45697; institutionCode: AAU
- **Type status:** Other material. **Occurrence:** recordedBy: Y. Ito; **Location:** country: Thailand; locality: Saraburi; verbatimLatitude: 14° 35' 10" N; verbatimLongitude: 100° 54' 32" E; **Event:** eventDate: Feb. 28, 1982; **Record Level:** collectionID: A. Ubolcholaket s.n.; institutionCode: AAU
- **Type status:** Other material. **Occurrence:** recordedBy: Y. Ito; **Location:** country: Thailand; locality: Naai Province; Tah Wang Pah District, Naha Han Village; verbatimLatitude: 19° 6' 44" N; verbatimLongitude: 100° 45' 16" E; **Event:** eventDate: Mar. 15, 2000; **Record Level:** collectionID: J.F. Maxwell 00-131; institutionCode: GH
- **Type status:** Other material. **Occurrence:** recordedBy: Y. Ito; **Location:** country: Thailand; locality: Thonburi; verbatimLatitude: 13° 45' N; verbatimLongitude: 100° 30' E; **Event:** eventDate: Nov. 15, 1970; **Record Level:** collectionID: J.F. Maxwell 70-85; institutionCode: AAU
- **Type status:** Other material. **Occurrence:** recordedBy: Y. Ito; **Location:** country: Thailand; locality: Ang Thong Province; Muang District; verbatimLatitude: 14° 35' 41" N; verbatimLongitude: 100° 27' 25" E; **Event:** eventDate: Dec. 11, 1971; **Record Level:** collectionID: J.F. Maxwell 71-787; institutionCode: AAU
- **Type status:** Other material. **Occurrence:** recordedBy: Y. Ito; **Location:** country: Thailand; locality: Saraburi Province; Muang District, Sahm Lahn forest; verbatimLatitude: 14° 31' 51" N; verbatimLongitude: 100° 54' 34" E; **Event:** eventDate: Jun. 30, 1974; **Record Level:** collectionID: J.F. Maxwell 74-648; institutionCode: AAU
- **Type status:** Other material. **Occurrence:** recordedBy: Y. Ito; **Location:** country: Thailand; locality: Songkla Province; Haad Yai District, Klong Hoy Kong, W of Toong Loong; verbatimLatitude: 7° 0' 13" N; verbatimLongitude: 100° 27' 25" e; **Event:** eventDate: May. 30, 1985; **Record Level:** collectionID: J.F. Maxwell 85-548; institutionCode: GH
- **Type status:** Other material. **Occurrence:** recordedBy: Y. Ito; **Location:** country: Thailand; locality: Songkla Province; Muang - Chana Districts Border; verbatimLatitude: 6° 59' 6" N; verbatimLongitude: 100° 39' 26" e; **Event:** eventDate: Jan. 17, 1985; **Record Level:** collectionID: J.F. Maxwell 85-75; institutionCode: GH
- **Type status:** Other material. **Occurrence:** recordedBy: Y. Ito; **Location:** country: Thailand; locality: Surattani Province; Ban Takun Distr., Klong Saeng Wildlife Sanctuary.; verbatimLatitude: 9° 9' 43" N; verbatimLongitude: 98° 53' 21" E; **Event:** eventDate: Feb. 16, 1994; **Record Level:** collectionID: J.F. Maxwell 94-230; institutionCode: GH
- **Type status:** Other material. **Occurrence:** recordedBy: Y. Ito; **Location:** country: Thailand; locality: N of pie Mai; **Event:** eventDate: Mar. 19, 1958; **Record Level:** collectionID: Th. Sorensen 2145; institutionCode: AAU
- **Type status:** Other material. **Occurrence:** recordedBy: Y. Ito; **Location:** country: Thailand; locality: Chaiyaphum Province; Thung Kra Mang; verbatimLatitude: 16° 15' N; verbatimLongitude: 101° 30' E; **Event:** eventDate: Aug. 9, 1972; **Record Level:** collectionID: K. Larsen et al. 31632; institutionCode: AAU
- **Type status:** Other material. **Occurrence:** recordedBy: Y. Ito; **Location:** country: Thailand; locality: Chanthaburi Province; Bor Rai50 km E of Makham; verbatimLatitude: 12° 52' N; verbatimLongitude: 102° 7' E; **Event:** eventDate: Sep. 1, 1972; **Record Level:** collectionID: K. Larsen et al. 32313; institutionCode: AAU
- **Type status:** Other material. **Occurrence:** recordedBy: Y. Ito; **Location:** country: Thailand; locality: Narathiwat Province; S of Naratiwat; verbatimLatitude: 6° 30' N; verbatimLongitude: 101° 45' E; **Event:** eventDate: Mar. 9, 1974; **Record Level:** collectionID: K. Larsen et al. 33129; institutionCode: AAU
- **Type status:** Other material. **Occurrence:** recordedBy: Y. Ito; **Location:** country: Thailand; locality: Nakhon Ratchasima Province; Pak Thong Chai District, E part of Khao Yai Natl Park; verbatimLatitude: 14° 22' N; verbatimLongitude: 101° 12' E; **Event:** eventDate: Aug. 11, 1968; **Record Level:** collectionID: K. Larsen et al. 3339; institutionCode: AAU
- **Type status:** Other material. **Occurrence:** recordedBy: Y. Ito; **Location:** country: Thailand; locality: Nakhon Ratchasima Province; Phimai; verbatimLatitude: 15° 5' N; verbatimLongitude: 101° 39' E; **Event:** eventDate: Jan. 12, 1967; **Record Level:** collectionID: T. Smitinand 33646; institutionCode: AAU
- **Type status:** Other material. **Occurrence:** recordedBy: Y. Ito; **Location:** country: Thailand; locality: Chiang Mai Province; San Sai District; verbatimLatitude: 18° 56' 29" N; verbatimLongitude: 99° 1' 48" E; **Event:** eventDate: Sep. 16, 1958; **Record Level:** collectionID: Th. Sorensen 5017; institutionCode: AAU
- **Type status:** Other material. **Occurrence:** recordedBy: Y. Ito; **Location:** country: Thailand; locality: Narathiwat Province; Kok Dam peat Swamp forest; verbatimLatitude: 6° 26' 17" N; verbatimLongitude: 101° 49' 26" E; **Event:** eventDate: Feb. 29, 1984; **Record Level:** collectionID: C. Niyomdham 807; institutionCode: AAU
- **Type status:** Other material. **Occurrence:** recordedBy: Y. Ito; **Location:** country: Thailand; locality: Chainat Province; ca. 10 miles W of ta Klee toward; verbatimLatitude: 15° 14' N; verbatimLongitude: 100° 15' E; **Event:** eventDate: Nov. 28, 1959; **Record Level:** collectionID: L. B. & E. C. Abbe, T. Smitinand 9232; institutionCode: GH
- **Type status:** Other material. **Occurrence:** recordedBy: Y. Ito; **Location:** country: Thailand; locality: Buri Ram Province; near Ban Kruat; verbatimLatitude: 14° 30' N; verbatimLongitude: 103° 7' E; **Event:** eventDate: Oct. 4, 1984; **Record Level:** collectionID: G. Murata, C. Phengklai, S. Mitsuta, H. Nagamasu, N. Nantasan T-37406; institutionCode: AAU
- **Type status:** Other material. **Occurrence:** recordedBy: Y. Ito; **Location:** country: Thailand; locality: Khao San Yot Natl Park.; verbatimLatitude: 12° 14' 40" N; verbatimLongitude: 99° 55' 58" E; **Event:** eventDate: Nov. 13, 2012; **Record Level:** collectionID: Y. Ito 1702; institutionCode: BKF
- **Type status:** Other material. **Occurrence:** recordedBy: Y. Ito; **Location:** country: Thailand; locality: Phetchabury.; verbatimLatitude: 13° 30' 6" N; verbatimLongitude: 99° 47' 37" E; **Event:** eventDate: Nov. 14, 2012; **Record Level:** collectionID: Y. Ito 1712; institutionCode: BKF
- **Type status:** Other material. **Occurrence:** recordedBy: Y. Ito; **Location:** country: Thailand; locality: Kantchanabury; verbatimLatitude: 14° 39' 21" N; verbatimLongitude: 98° 42' 27" E; **Event:** eventDate: Nov. 15, 2012; **Record Level:** collectionID: Y. Ito 1730; institutionCode: BKF

##### Distribution

Bangladesh, China (Central, South), India (nationwide), Japan, Malesia (throughout), Nepal, Pakistan; Oceania.

#### Utricularia australis

R. Br., 1810

##### Materials

- **Type status:** Other material. **Occurrence:** recordedBy: Y. Ito; **Location:** country: Myanmar; locality: Bago Division, Donese; verbatimLatitude: 17° 37' 6'' N; verbatimLongitude: 95° 47' 22'' E; **Event:** eventDate: Dec. 8, 2006; **Record Level:** collectionID: Sugawara et al. 036321; institutionCode: TI
- **Type status:** Other material. **Occurrence:** recordedBy: Y. Ito; **Location:** country: Myanmar; locality: Shan State; Pindaya Township, Inya lake; verbatimLatitude: 20° 59' 57'' N; verbatimLongitude: 96° 39' 59'' E; **Event:** eventDate: Dec. 1, 2008; **Record Level:** collectionID: Tanaka et al. 080627; institutionCode: TI
- **Type status:** Other material. **Occurrence:** recordedBy: Y. Ito; **Location:** country: Myanmar; locality: Shan State; Pindaya Township, Inlay lake; verbatimLatitude: 20° 32' 2'' N; verbatimLongitude: 96° 53' 53'' E; **Event:** eventDate: Dec. 3, 2008; **Record Level:** collectionID: Tanaka et al. 080644; institutionCode: TI
- **Type status:** Other material. **Occurrence:** recordedBy: Y. Ito; **Location:** country: Myanmar; locality: Shan State; Nyaung Shwe Towhship, Inlay Lake; verbatimLatitude: 20° 32' 2'' N; verbatimLongitude: 96° 53' 53'' E; **Event:** eventDate: Dec. 3, 2008; **Record Level:** collectionID: Tanaka et al. 080659; institutionCode: TI
- **Type status:** Other material. **Occurrence:** recordedBy: Y. Ito; **Location:** country: Myanmar; locality: Mandaley Region; Maymyo (Pyinoolwin) Plateau; verbatimLatitude: 22° 2' 12" N; verbatimLongitude: 96° 28' 10" E; **Event:** eventDate: Jan. 9, 1912; **Record Level:** collectionID: J.H. Lace 5948; institutionCode: GH
- **Type status:** Other material. **Occurrence:** recordedBy: Y. Ito; **Location:** country: Myanmar; locality: Mandaley Region; Maymyo (Pyinoolwin); verbatimLatitude: 22° 2' 12" N; verbatimLongitude: 96° 28' 10" E; **Event:** eventDate: Jan. 4, 1933; **Record Level:** collectionID: F.G. Dickason 5994; institutionCode: GH

##### Distribution

Bhutan, China (nationwide), India (nationwide), Indonesia (Java, Sumatra), Japan, Nepal, Pakistan, Papua New Guinea, Philippines, Sri Lanka; Africa, Europe, Oceania.

#### Utricularia gibba

L., 1753

##### Materials

- **Type status:** Other material. **Occurrence:** recordedBy: Y. Ito; **Location:** country: Myanmar; locality: Shan State; Inlay lake, Nyanug She Township; verbatimLatitude: 16° 53' 19" N; verbatimLongitude: 95° 52' 29" E; **Event:** eventDate: Dec. 3, 2008; **Record Level:** collectionID: Tanaka et al. 080639; institutionCode: TI
- **Type status:** Other material. **Occurrence:** recordedBy: Y. Ito; **Location:** country: Myanmar; locality: Tenasserim Division, Tawer District, Yebyu Township, Kan Bank Village area, coastal region; verbatimLatitude: 12° 5' 11" N; verbatimLongitude: 99° 0' 51" E; **Event:** eventDate: Mar. 2, 1996; **Record Level:** collectionID: J.F. Maxwell 96-335; institutionCode: BKF
- **Type status:** Other material. **Occurrence:** recordedBy: Y. Ito; **Location:** country: Thailand; locality: Songkla Province; Talae Noi waterfowl reserve; verbatimLatitude: 7° 15' N; verbatimLongitude: 100° 26' 16" E; **Event:** eventDate: Dec. 28, 1978; **Record Level:** collectionID: G. Congdon, C. Hamilton 159; institutionCode: AAU
- **Type status:** Other material. **Occurrence:** recordedBy: Y. Ito; **Location:** country: Thailand; locality: Lopburi Province; Supcham Pa Hill; verbatimLatitude: 14° 50' N; verbatimLongitude: 100° 55' E; **Event:** eventDate: Nov. 19, 1984; **Record Level:** collectionID: G. Murata, C. Phengklai, S. Mitsuta, T. Yahara, H. Nagamasu, N. Nantasan T-50927; institutionCode: BKF
- **Type status:** Other material. **Occurrence:** recordedBy: Y. Ito; **Location:** country: Thailand; locality: Chiang Mai Province; Doi Inthanon, near guest house; verbatimLatitude: 18° 32' 55" N; verbatimLongitude: 98° 31' 29" E; **Event:** eventDate: Jul. 23, 1988; **Record Level:** collectionID: S. Tsugaru T-61738; institutionCode: BKF
- **Type status:** Other material. **Occurrence:** recordedBy: Y. Ito; **Location:** country: Thailand; locality: Thathungna Dam, Kantchanabury; verbatimLatitude: 14° 13' 53" N; verbatimLongitude: 99° 13' 44" E; **Event:** eventDate: Nov. 15, 2012; **Record Level:** collectionID: Y. Ito 1725; institutionCode: BKF

##### Distribution

Bangladesh, China (nationwide), India (nationwide), Indonesia (nationwide), Japan, Malaysia (nationwide), Myanmar, Nepal, Papua New Guinea, Sri Lanka, Thailand, ?Vietnam; Africa; Oceania; N. America; S. America.

#### Utricularia punctata

Wall. ex A. DC., 1844

##### Materials

- **Type status:** Other material. **Occurrence:** recordedBy: Y. Ito; **Location:** country: Myanmar; locality: Tenasserim Division, Tawer District, Yebyu Township, Kan Bank Village area, coastal region; verbatimLatitude: 12° 5' 11" N; verbatimLongitude: 99° 0' 51" E; **Event:** eventDate: Mar. 2, 1996; **Record Level:** collectionID: J.F. Maxwell 96-336; institutionCode: BKF
- **Type status:** Other material. **Occurrence:** recordedBy: Y. Ito; **Location:** country: Thailand; locality: Phang-nga Province; Kuraburi District, Bangwan Stream; verbatimLatitude: 9° 13' 34" N; verbatimLongitude: 98° 26' 22" E; **Event:** eventDate: Dec. 28, 2006; **Record Level:** collectionID: T. Muadsud 210; institutionCode: BKF
- **Type status:** Other material. **Occurrence:** recordedBy: Y. Ito; **Location:** country: Thailand; locality: Narathiwat Province; Bang Nara River; verbatimLatitude: 6° 23' 20" N; verbatimLongitude: 101° 49' 26" E; **Event:** eventDate: Jan. 20, 2000; **Record Level:** collectionID: C. Niyomdham et al. 5990; institutionCode: BKF

##### Distribution

China (South), Indonesia (Sumatra), Malaysia (Borneo, Malaya), Myanmar, Thailand, ?Vietnam.

#### Utricularia stellaris

L. f., 1781

##### Materials

- **Type status:** Other material. **Occurrence:** recordedBy: Y. Ito; **Location:** country: Myanmar; locality: Rangoon; verbatimLatitude: 16° 49' 37" N; verbatimLongitude: 96° 8' 58" E; **Event:** eventDate: Jan. 2, 1938; **Record Level:** collectionID: F.G. Dickason 6965; institutionCode: GH
- **Type status:** Other material. **Occurrence:** recordedBy: Y. Ito; **Location:** country: Myanmar; locality: Bago Division; Pyat Township.; verbatimLatitude: 18° 49' 44'' N; verbatimLongitude: 95° 18' 6'' E; **Event:** eventDate: Dec. 7, 2008; **Record Level:** collectionID: Tanaka et al. 080765; institutionCode: TI

##### Distribution

Bangladesh, India (nationwide), Myanmar, Nepal, Sri lanka; Africa.

##### Notes

Fig. 10.

#### Asterales

#### Menyanthaceae

#### Nymphoides

Ség., 1754

#### Nymphoides aurantiaca

(Dalzell) Kuntze, 1891

##### Materials

- **Type status:** Other material. **Occurrence:** recordedBy: Y. Ito; **Location:** country: Thailand; locality: Krabi province; Ban Sai Khao, Konglom; verbatimLatitude: 7° 44' 59" N; verbatimLongitude: 99° 15' 48" E; **Event:** eventDate: Oct. 1, 1960; **Record Level:** collectionID: ???; institutionCode: TI
- **Type status:** Other material. **Occurrence:** recordedBy: Y. Ito; **Location:** country: Thailand; locality: Surat Thani Province; Tochang District, Ban Phace, Elong Sai Subdistrict; verbatimLatitude: 9° 18' 15" N; verbatimLongitude: 99° 3' 19" E; **Event:** eventDate: Feb. 5, 1987; **Record Level:** collectionID: E.P. 1246; institutionCode: BKF
- **Type status:** Other material. **Occurrence:** recordedBy: Y. Ito; **Location:** country: Thailand; locality: Chanthabury Province; 18 km W of Trak; verbatimLatitude: 12° 57' N; verbatimLongitude: 102° 0' E; **Event:** eventDate: Oct. 23, 1972; **Record Level:** collectionID: No. 2027; institutionCode: BKF
- **Type status:** Other material. **Occurrence:** recordedBy: Y. Ito; **Location:** country: Thailand; locality: Surin; verbatimLatitude: 14° 55' 53" N; verbatimLongitude: 103° 29' 17" E; **Event:** eventDate: Nov. 20, 1976; **Record Level:** collectionID: J.F. Maxwell 72-531; institutionCode: AAU
- **Type status:** Other material. **Occurrence:** recordedBy: Y. Ito; **Location:** country: Thailand; locality: Ranong Province; Ban phe; verbatimLatitude: 12° 40' N; verbatimLongitude: 101° 25' E; **Event:** eventDate: Dec. 16, 1974; **Record Level:** collectionID: C. Phengklai et al. 3303; institutionCode: BKF, GH
- **Type status:** Other material. **Occurrence:** recordedBy: Y. Ito; **Location:** country: Thailand; locality: Ranong Province; near the town; verbatimLatitude: 9° 57' 14" N; verbatimLongitude: 98° 36' 22" E; **Event:** eventDate: Nov. 23, 1970; **Record Level:** collectionID: R. Geesink, P. Hiepko 7871; institutionCode: BKF
- **Type status:** Other material. **Occurrence:** recordedBy: Y. Ito; **Location:** country: Thailand; locality: Surattani Province; Tachang Ban Tahsae District, Klong Sai sub-district; verbatimLatitude: 9° 17' 58" N; verbatimLongitude: 99° 3' 27" E; **Event:** eventDate: Feb. 5, 1987; **Record Level:** collectionID: T. Smitinand et al. 11591; institutionCode: BKF

##### Distribution

India (Southern), Papua New Guinea, Thailand; Oceania.

##### Notes

Fig. 11.

#### Nymphoides cambodiana

(Hance) Tippery, 2009

##### Materials

- **Type status:** Other material. **Occurrence:** recordedBy: Y. Ito; **Location:** country: Thailand; locality: Roi-et Province; verbatimLatitude: 16° 2' N; verbatimLongitude: 103° 38' E; **Event:** eventDate: Jun. 22, 1969; **Record Level:** collectionID: J.F. Maxwell 87-165; institutionCode: GH
- **Type status:** Other material. **Occurrence:** recordedBy: Y. Ito; **Location:** country: Thailand; locality: Nakhon Ratchasima Province; verbatimLatitude: 14° 36' N; verbatimLongitude: 101° 33' E; **Event:** eventDate: Feb. 11, 1975; **Record Level:** collectionID: No. 46634; institutionCode: BKF

##### Distribution

Cambodia, Laos, Thailand, Vietnam.

#### Nymphoides hydrophylla

(Lour.) Kuntze, 1891

##### Materials

- **Type status:** Other material. **Occurrence:** recordedBy: Y. Ito; **Location:** country: Myanmar; locality: Shan State; Inlay lake, Nyanug She Township; verbatimLatitude: 20° 32' 2" N; verbatimLongitude: 96° 53' 53" E; **Event:** eventDate: Dec. 3, 2008; **Record Level:** collectionID: A. Ubolcholaket s.n.; institutionCode: AAU
- **Type status:** Other material. **Occurrence:** recordedBy: Y. Ito; **Location:** country: Myanmar; locality: Kachin State; S of Tanaing, Hukaung Valley; verbatimLatitude: 26° 06' 34" N; verbatimLongitude: 96° 42' 58" E; **Event:** eventDate: Sep. 19, 2005; **Record Level:** collectionID: Nb. Tanaka et al. 080654; institutionCode: TI
- **Type status:** Other material. **Occurrence:** recordedBy: Y. Ito; **Location:** country: Thailand; locality: Songkhla Province; Songkhla - Pattani road, ca 50km from Songkhla; verbatimLatitude: 6° 53' N; verbatimLongitude: 100° 55' E; **Event:** eventDate: Oct. 31, 1990; **Record Level:** collectionID: Nb. Tanaka et al. 040480; institutionCode: TI
- **Type status:** Other material. **Occurrence:** recordedBy: Y. Ito; **Location:** country: Thailand; locality: Chanthabury Province; between Chanthaburi and Trat; verbatimLatitude: 12° 40' N; verbatimLongitude: 102° 20' E; **Event:** eventDate: Sep. 3, 1972; **Record Level:** collectionID: K. Larsen et al. 41006; institutionCode: AAU
- **Type status:** Other material. **Occurrence:** recordedBy: Y. Ito; **Location:** country: Thailand; locality: Loei Province; Khunnaamphong, Phukradueng Natl Park; verbatimLatitude: 16° 54' N; verbatimLongitude: 101° 45' E; **Event:** eventDate: Sep. 8, 1988; **Record Level:** collectionID: K. Larsen et al. 32398; institutionCode: AAU
- **Type status:** Other material. **Occurrence:** recordedBy: Y. Ito; **Location:** country: Thailand; locality: Narathiwat; verbatimLatitude: 6° 26' N; verbatimLongitude: 101° 49' E; **Event:** eventDate: Sep. 18, 1965; **Record Level:** collectionID: R. Pooma 85; institutionCode: BKF
- **Type status:** Other material. **Occurrence:** recordedBy: Y. Ito; **Location:** country: Thailand; locality: Ranong Province; Bang Ben, Laem Son Forest Park; verbatimLatitude: 9° 28' N; verbatimLongitude: 98° 23' E; **Event:** eventDate: Sep. 10, 1982; **Record Level:** collectionID: Chamlong 1104; institutionCode: BKF

##### Distribution

Cambodia, China (Southern [Taiwan]), Laos, Myanmar, Thailand, Vietnam.

##### Notes

Fig. 12.

#### Nymphoides indica

(L.) Kuntze, 1891

##### Materials

- **Type status:** Other material. **Occurrence:** recordedBy: Y. Ito; **Location:** country: Myanmar; locality: Bago Division; W of Pyay; verbatimLatitude: 18° 42' 22" N; verbatimLongitude: 95° 5' 59" E; **Event:** eventDate: Dec. 9, 2006; **Record Level:** collectionID: T. Shimizu et al. T-29263; institutionCode: BKF
- **Type status:** Other material. **Occurrence:** recordedBy: Y. Ito; **Location:** country: Myanmar; locality: Rakhain State; Linthar, Ngapali Dam; verbatimLatitude: 19° 24' 27" N; verbatimLongitude: 94° 20' 3" E; **Event:** eventDate: Dec. 12, 2006; **Record Level:** collectionID: T. Sugawara et al. 036430; institutionCode: TI
- **Type status:** Other material. **Occurrence:** recordedBy: Y. Ito; **Location:** country: Myanmar; locality: Shan State; Inya lake, N of Pindaya; verbatimLatitude: 20° 59' 57" N; verbatimLongitude: 96° 39' 59" E; **Event:** eventDate: Dec. 1, 2008; **Record Level:** collectionID: T. Sugawara et al. 036591; institutionCode: TI
- **Type status:** Other material. **Occurrence:** recordedBy: Y. Ito; **Location:** country: Myanmar; locality: Shan State; Inlaylake, Nyaung Shwe Township; verbatimLatitude: 20° 32' 2" N; verbatimLongitude: 96° 53' 53" E; **Event:** eventDate: Dec. 3, 2008; **Record Level:** collectionID: Nb. Tanaka et al. 080629; institutionCode: TI
- **Type status:** Other material. **Occurrence:** recordedBy: Y. Ito; **Location:** country: Thailand; locality: Chiang Mai Province; Northern District, along road to San Kamphaeng, E of Chiang Mai; verbatimLatitude: 18° 47' 16" N; verbatimLongitude: 99° 0' 39" E; **Event:** eventDate: Jun. 17, 1968; **Record Level:** collectionID: Nb. Tanaka et al. 080643; institutionCode: TI
- **Type status:** Other material. **Occurrence:** recordedBy: Y. Ito; **Location:** country: Thailand; locality: Chanthabury Province; 18 km W of Trak; verbatimLatitude: 12° 57' N; verbatimLongitude: 102° 0' E; **Event:** eventDate: Oct. 2, 1972; **Record Level:** collectionID: C.F. van Beusekom, C. Phengkhlai 1302; institutionCode: AAU
- **Type status:** Other material. **Occurrence:** recordedBy: Y. Ito; **Location:** country: Thailand; locality: Songkla Province; Muang - Chana Districts Border; verbatimLatitude: 6° 59' 6" N; verbatimLongitude: 100° 39' 26" E; **Event:** eventDate: Jan. 17, 1985; **Record Level:** collectionID: J.F. Maxwell 72-532; institutionCode: AAU
- **Type status:** Other material. **Occurrence:** recordedBy: Y. Ito; **Location:** country: Thailand; locality: Songla Province; Talae Noi Waterfowl Reserve, N end of Lake Songkla, near Phattalung; verbatimLatitude: 7° 15' N; verbatimLongitude: 100° 26' 16" E; **Event:** eventDate: Dec. 28, 1978; **Record Level:** collectionID: J.F. Maxwell 85-74; institutionCode: GH
- **Type status:** Other material. **Occurrence:** recordedBy: Y. Ito; **Location:** country: Thailand; locality: Narathiwat Province; Paa Waai, Su Ngi Paadee; verbatimLatitude: 6° 7' 37" N; verbatimLongitude: 101° 54' 52" E; **Event:** eventDate: Sep. 4, 1987; **Record Level:** collectionID: G. Congdon, C. Hamilton 153; institutionCode: AAU
- **Type status:** Other material. **Occurrence:** recordedBy: Y. Ito; **Location:** country: Thailand; locality: Surin; verbatimLatitude: 14° 55' 53" N; verbatimLongitude: 103° 29' 17" E; **Event:** eventDate: Nov. 20, 1976; **Record Level:** collectionID: C. Niyomdham & D. Sriboonma 1544; institutionCode: AAU
- **Type status:** Other material. **Occurrence:** recordedBy: Y. Ito; **Location:** country: Thailand; locality: Nakhon Nayok Province; verbatimLatitude: 14° 12' 17" N; verbatimLongitude: 101° 13' 36" E; **Event:** eventDate: Aug. 6, 1970; **Record Level:** collectionID: C. Phengklai et al. 3304; institutionCode: GH
- **Type status:** Other material. **Occurrence:** recordedBy: Y. Ito; **Location:** country: Thailand; locality: Phitsanulok Province; Tung Salaeng Luang; verbatimLatitude: 16° 59' N; verbatimLongitude: 100°53' E; **Event:** eventDate: Jul. 19, 1966; **Record Level:** collectionID: C. Phengklai et al. 3750; institutionCode: GH
- **Type status:** Other material. **Occurrence:** recordedBy: Y. Ito; **Location:** country: Thailand; locality: East, Si Saket Prov.; verbatimLatitude: 15° 7' N; verbatimLongitude: 104° 15' E; **Event:** eventDate: Oct. 7, 1984; **Record Level:** collectionID: K. Larsen et al. 512; institutionCode: AAU
- **Type status:** Other material. **Occurrence:** recordedBy: Y. Ito; **Location:** country: Thailand; locality: Kantchanabury Province; Hotel river Kwai; verbatimLatitude: 14° 1' 59" N; verbatimLongitude: 99° 31' 10" E; **Event:** eventDate: Nov. 15, 2012; **Record Level:** collectionID: G. Murata, C. Phengklai, S. Mitsuta, H. Nagamasu, N. Nantasan T-49919; institutionCode: AAU, GH
- **Type status:** Other material. **Occurrence:** recordedBy: Y. Ito; **Location:** country: Thailand; locality: Surin; verbatimLatitude: 14° 55' 53" N; verbatimLongitude: 103° 29' 17" E; **Event:** eventDate: Dec. 8, 1976; **Record Level:** collectionID: Y. Ito 1718; institutionCode: BKF

##### Distribution

Bangladesh, ?Cambodia, China, India (nationwide), ?Indonesia, Japan, ?Malaysia, Myanmar, Nepal, Papua New Guinea, Sri Lanka, ?Vietnam; Oceania; N. America; S. America.

## Analysis

The 78 species were classified into six geographic categories: Widespread; Eurasian cool-temperate; Asia – Australia; Tropical Asia; Pan-Tropics; invasive (Table 2). The data concerning the general distribution was taken Boyce (2012), Boyce et al. (2012), Chayamarit (2005), Cook (1996), Guo et al. (2010), Haynes (2001f), Haynes (2001d), Haynes (2001a), Haynes (2001e), Haynes (2001b), Haynes (2001c), Ho and Ornduff (1995), Ito (2013b), Ito et al. (2009), Landolt (2001), Simpson (2008), Larsen (2000), Tippery et al. (2009), Yamazaki (1990), Barkera and Jobson (2013).

## Discussion

Altogether, we identified 380 specimens, excluding 31 duplicates, belonging to 75 species of 41 genera from 23 plant families; the remaining 3 species belonging to 3 genera of 3 families were listed with no voucher specimens. Geographically the species distribution pattern can be classified into six categories: Widespread; Eurasian cool-temperate; Asia – Australia; Tropical Asia; Pan-Tropics; and Invasive.

### Widespread

This category is mostly congruent with 'Widespread Species' of Sculthorpe (1967).

### Eurasian cool-temperate

The species clustered in this category are distributed mainly in cool-temperate region of Eurasia, thus limiting the distribution to a small portion of Southeast Asia (Myanmar: Shan state; Thailand: Northern states).

### Asia – Australia

The species included in this category have a distribution in either Australia or East Asia or both, e.g., Australia: *Mimulus orbicularis* and *Nymphoides aurantiaca*; East Asia: *Acorus gramineus*, *Blyxa japonica*, *Limnophila sessiliflora*, *Potamogeton distinctus*, *Potamogeton maackianus*, *Potamogeton wrightii*, and *Sagittaria trifolia*; Australia and East Asia: *Blyxa aubertii*, *Blyxa echinosperma*, *Hydrocharis dubia*, *Limnophila indica*, *Monochoria vaginalis*, *Najas graminea*, *Ottelia alismoides*, Persicaria attenuata subsp. pulchra, *Potamogeton octandrus*, *Utricularia aurea*, and *Utricularia australis*.

### Asian tropics

This category comprises the largest number of species for the aquatic floras of Myanmar and Thailand. Some of the species included are endemic or narrowly distributed e.g., *Cryptocoryne* is endemic to tropical Asia. The monotypic genus *Nechamandra* occurs predominantly in India but also scattered in other parts of tropical Asia (also reported from one locality of Africa). Another monotypic genus *Barclaya* is among the tropical Asian endemics. The genera *Aponogeton* (Aponogetonaceae), *Blyxa*, *Najas*, (Hydrocharitaceae), *Myriophyllum* (Haloragaceae), *Nymphoides* (Menyanthaceae), *Monochoria* (Pontederiaceae), *Utricularia* (Lentibulariaceae) have their center of diversity in tropical Asia.

On a species level, the distribution is further classified into three groups: 1) Indo-China, 2) Southeast Asian, and 3) endemic to Myanmar and Thailand. The first group includes *Cryptocoryne crispatula*, *Eriocaulon setaceum*, *Limnophila heterophylla*, *Monochoria hastata*, *Myriophyllum tetrandrum*, *Myriophyllum tuberculatum*, *Najas indica*, *Najas graminea*, *Nechamandra alternifolia*, *Nymphaea nouchali*, *Nymphaea pubescens*, *Nymphoides hydrophylla*, and *Utricularia stellaris* (also known from Africa). The second group includes *Barclaya longifolia*, *Cryptocoryne albida*, *Cryptocoryne cordata*, *Monochoria elata*, and *Ottelia cordata*. The last group includes *Blyxa quadricostata*, *Crinum thaianum*, *Cryptocoryne cruddasiana*, and *Utricularia punctata*.

### Pan-tropics

This category with eight species is mostly congruent with 'Pan-tropical Species' of Sculthorpe (1967).

### Invasive

The aquatic floras of Myanmar and Thailand have well known invasive aquatic species including *Elodea densa* (Hydrocharitaceae) and *Eichhornia crassipes* (Pontederiaceae).

## Figures and Tables

**Figure 2.**
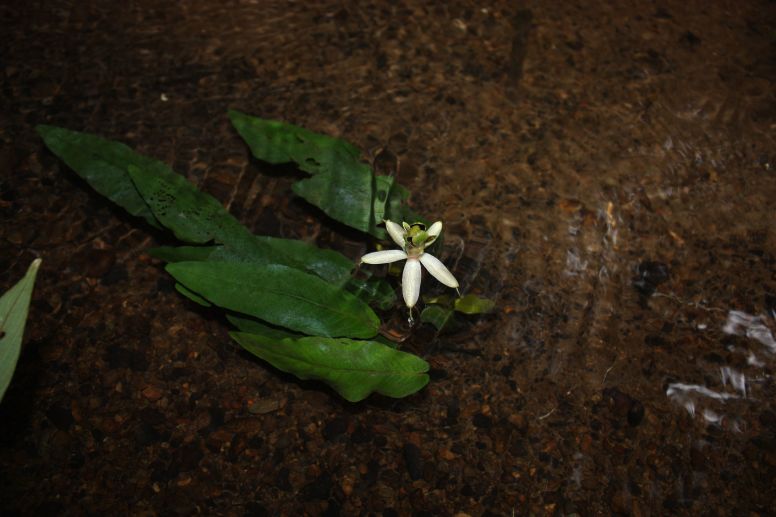
*Barclaya longifolia* in Thailand (25 Aug. 2010). Credit: R. Pooma.

**Figure 3.**
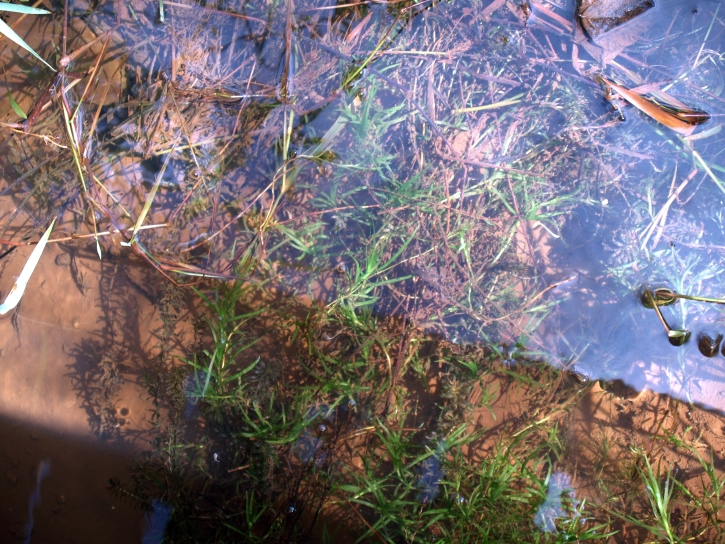
*Najas tenuis* in Myanmar (3 DEC 2008). Credit: Y. Ito.

**Figure 4.**
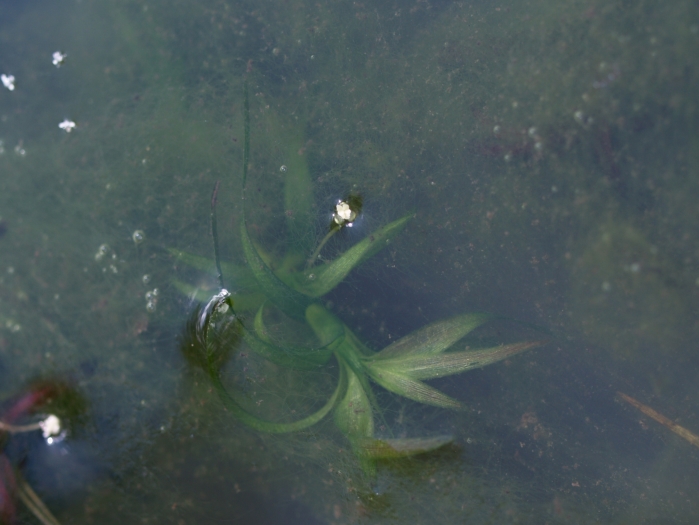
*Nechamandra alternifolia* (Hydrocharitaceae) (25 Nov. 2008). Credit: Y. Ito.

**Figure 5.**
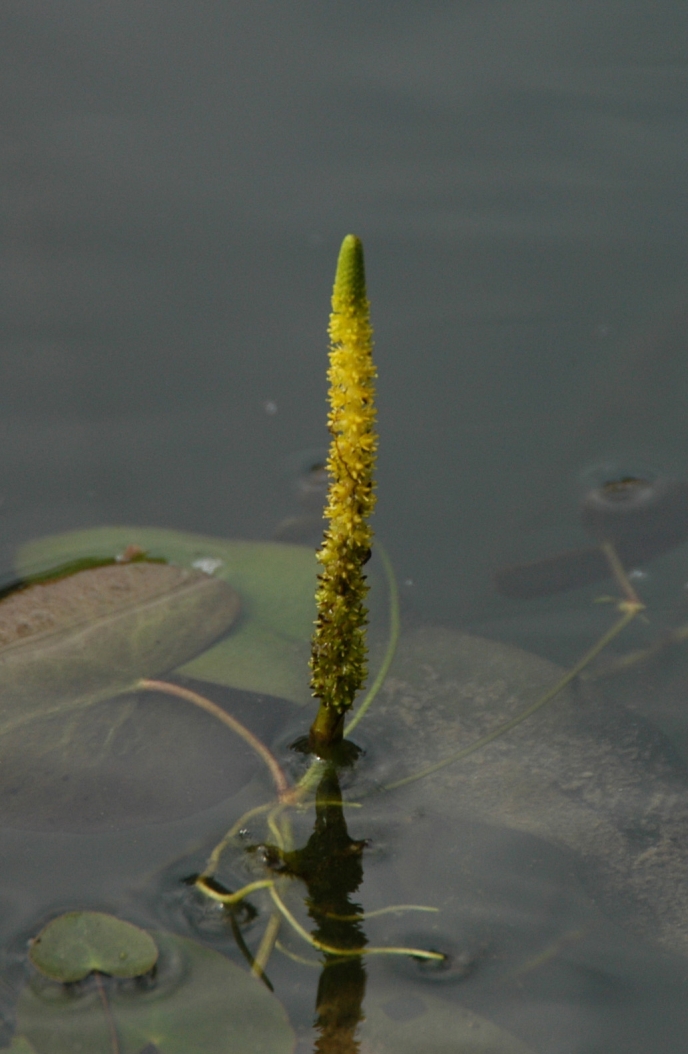
*Aponogeton lakhonensis* in Myanmar (10 Dec 2005). Credit: J. Murata.

**Figure 6.**
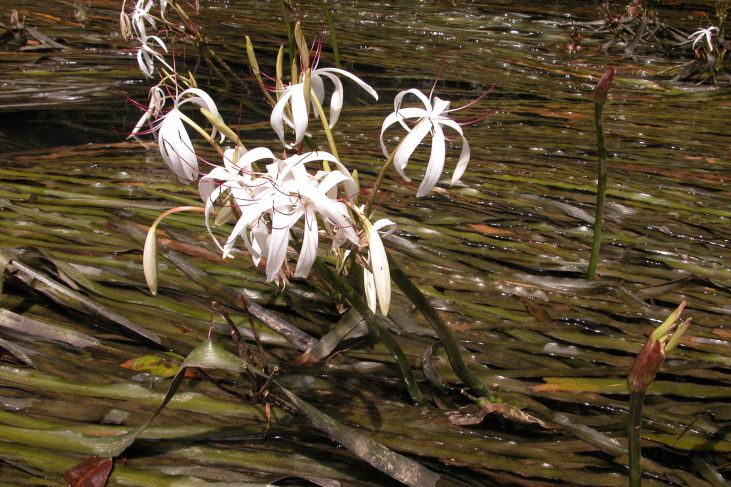
*Crinum thaianum* in Thailand (3 Dec. 2003). Credit: R. Pooma.

**Figure 7.**
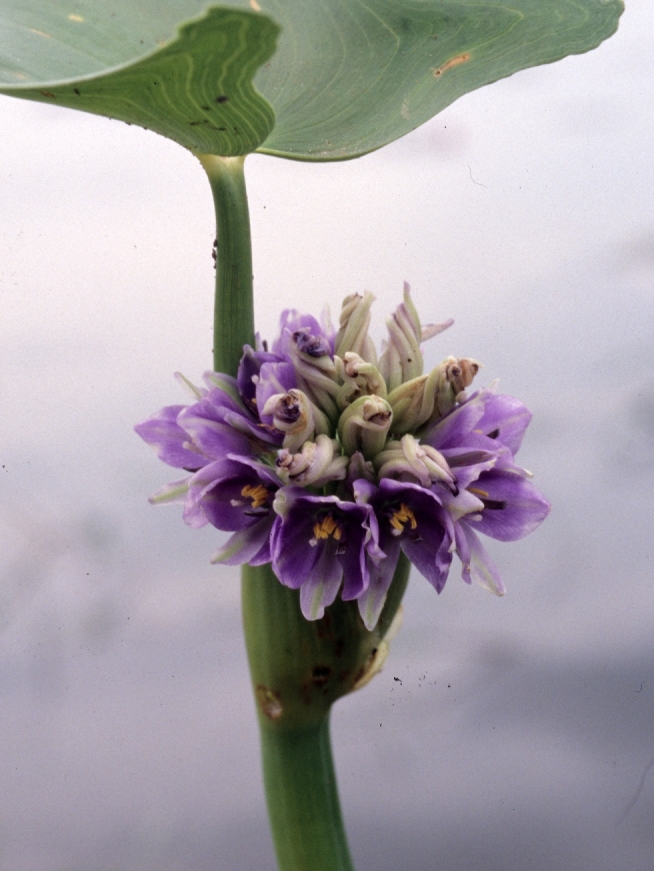
*Monochoria hastata* in Thailand (5 Sep. 2008). Credit: Udon.

**Figure 8.**
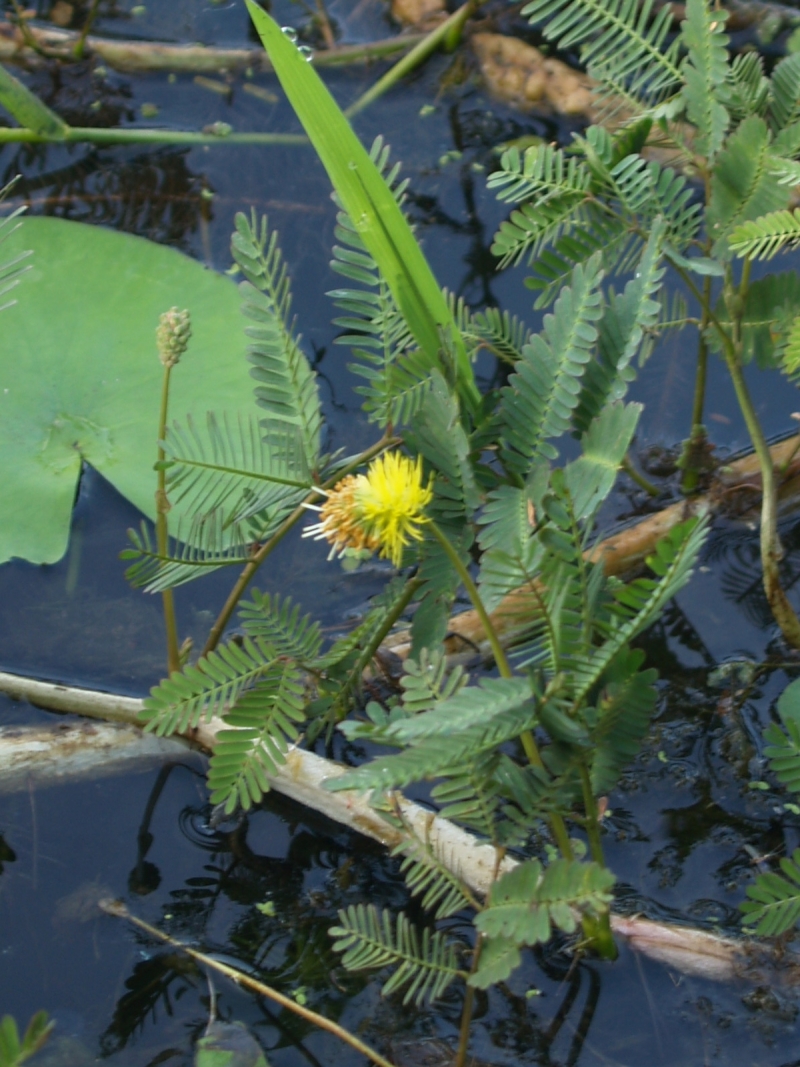
*Neptunia oleracea* in Thailand (15 Nov 2012). Credit: Y. Ito.

**Figure 9.**
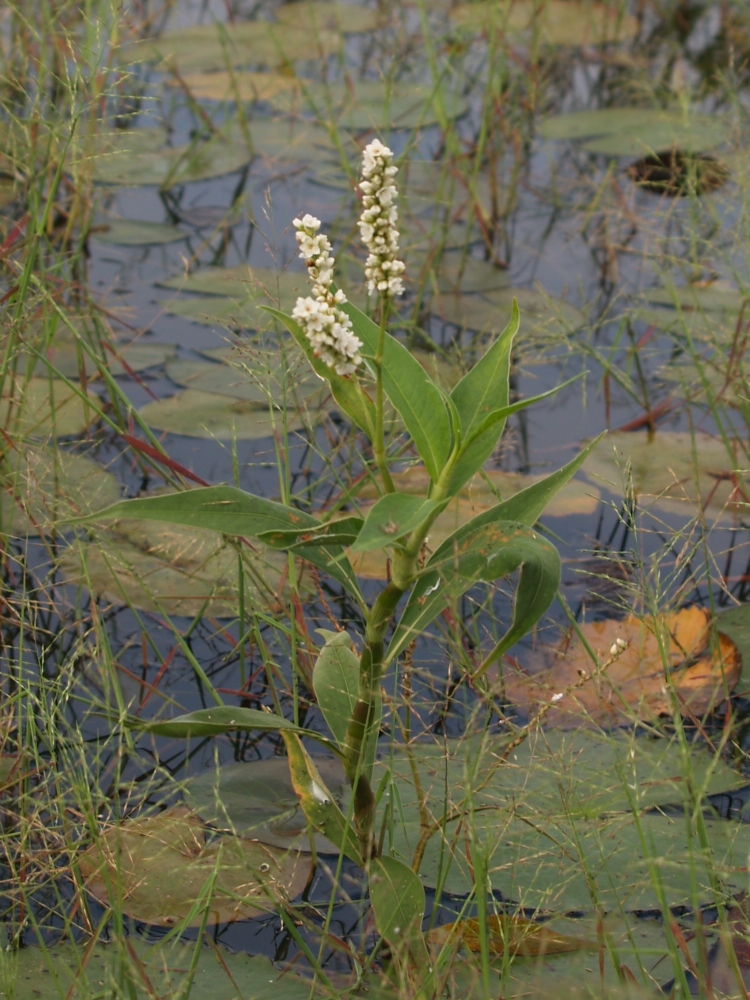
Persicaria attenuata subsp. pulchra in Thailand (17 Nov 2012). Credit: Y. Ito.

**Figure 10.**
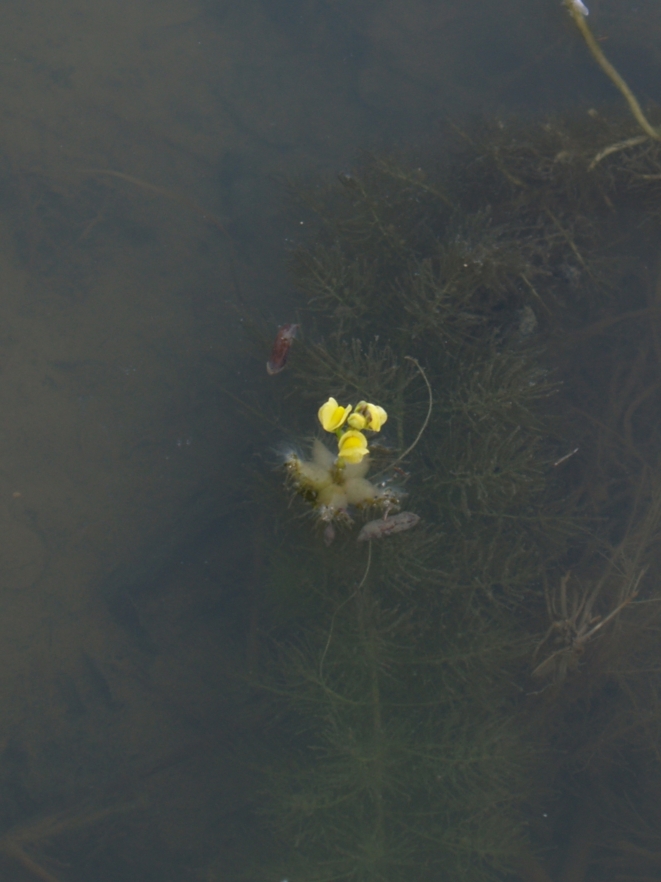
*Utricularia stellaris* in Myanmar (7 Dec 2008). Credit: Y. Ito.

**Figure 11.**
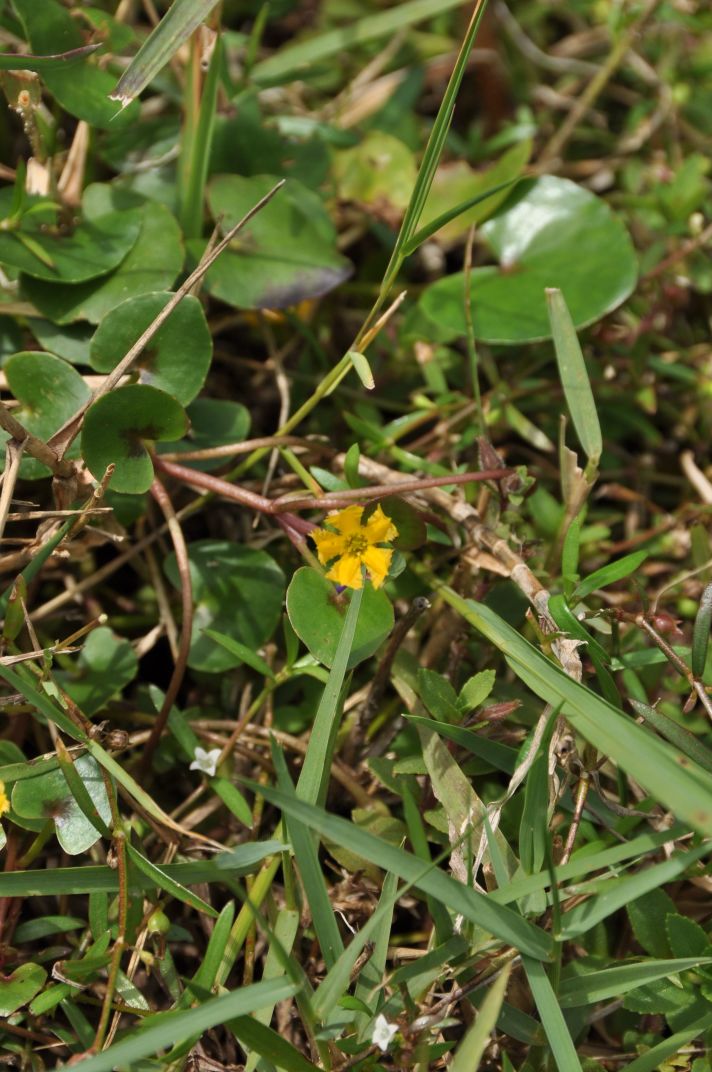
*Nymphoides aurantiaca* in Thailand (15 May 2000). Credit: R. Pooma.

**Figure 12.**
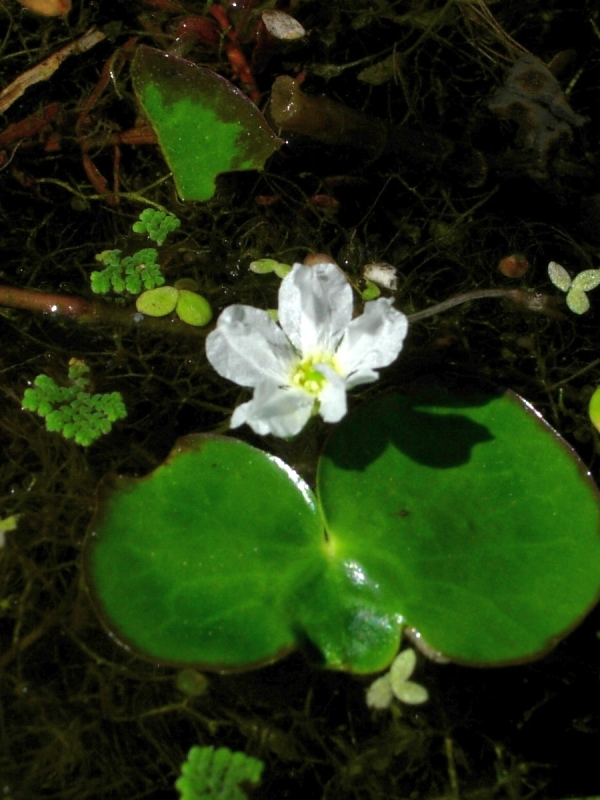
*Nymphoides hydrophylla* in Myanmar (3 Dec 2008). Credit: Y. Ito.

**Table 1.** Aquatic plant families treated in volumes of Flora of Thailand.

Volume	Family	Reference
Volume 5 part 2	Scrophulariaceae	Yamazaki (1990)
Volume 7 part 2	Callitrichaceae	Larsen (2000)
Volume 7 part 3	Alismataceae	Haynes (2001d)
Volume 7 part 3	Aponogetonaceae	Haynes (2001a)
Volume 7 part 3	Cymodoceaceae	Haynes (2001e)
Volume 7 part 3	Hydrocharitaceae	Haynes (2001b)
Volume 7 part 3	Lemnaceae	Landolt (2001)
Volume 7 part 3	Limnocharitaceae	Haynes (2001c)
Volume 7 part 3	Potamogetonaceae	Haynes (2001f)
Volume 9 part 1	Pontederiaceae	Chayamarit (2005)
Volume 9 part 2	Typhaceae	Simpson (2008)
Volume 11 part 2	Acoraceae	Boyce (2012)
Volume 11 part 2	Araceae	Boyce et al. (2012)
Volume 12 part 2	Ruppiaceae	Ito (2013a)

**Table 2.** A classification of aquatic plants in Myanmar and Thailand in distribution.

Widespread	Eurasian cool-temperate	Asia – Australia	Asian tropics	Pan-Tropics	Invasive
*Acorus calamus*	*Alisma plantago-aquatica*	*Acorus gramineus*	*Aponogeton lakhonensis*	*Hydrilla verticillata*	*Cabomba caroliniana*
*Caldesia parnassifolia*	*Potamogeton lucens*	*Blyxa aubertii*	*Barclaya longifolia*	*Ipomoea aquatica*	*Egeria densa*
*Callitriche stagnalis*	*Vallisneria spiralis*	*Blyxa echinosperma*	*Blyxa quadricostata*	*Lemna aequinoctialis*	*Eichhornia crassipes*
*Ceratophyllum demersum*		*Blyxa japonica*	*Crinum thaianum*	*Ludwigia adscendens*	*Elodea nutallii*
*Landoltia punctata*		*Hydrocharis dubia*	*Cryptocoryne albida*	*Neptunia oleracea*	*Limnocharis flava*
*Lemna trisulca*		*Limnophila indica*	*Cryptocoryne cordata*	*Nymphoides indica*	*Myriophyllum brasiliense*
*Najas marina*		*Limnophila sessiliflora*	*Cryptocoryne crispatula*	*Pistia stratiotes*	*Myriophyllum spicatum*
*Potamogeton crispus*		*Monochoria vaginalis*	*Cryptocoryne cruddasiana*	*Utricularia gibba*	*Nelumbo nucifera*
*Potamogeton nodosus*		*Mimulus orbicularis*	*Eriocaulon setaceum*		*Sagittaria guayanensis*
*Ruppia maritima*		*Nymphoides aurantiaca*	*Limnophila heterophylla*		
*Spirodela polyrrhiza*		*Najas graminea*	*Monochoria elata*		
*Stuckenia pectinata*		*Ottelia alismoides*	*Monochoria hastata*		
*Typha angustifolia*		Persicaria attenuata subsp. pulchra	*Myriophyllum tetrandrum*		
		*Potamogeton distinctus*	*Myriophyllum tuberculatum*		
		*Potamogeton maackianus*	*Najas indica*		
		*Potamogeton octandrus*	*Najas tenuis*		
		*Potamogeton wrightii*	*Nechamandra alternifolia*		
		*Sagittaria trifolia*	*Nymphaea nouchali*		
		*Utricularia aurea*	*Nymphaea pubescens*		
		*Utricularia australis*	*Nymphoides cambodiana*		
			*Nymphoides hydrophylla*		
			*Ottelia cordata*		
			*Utricularia punctata*		
			*Utricularia stellaris*		
			*Wolffia globosa*		
